# A Lignocellulolytic *Colletotrichum* sp. OH with Broad-Spectrum Tolerance to Lignocellulosic Pretreatment Compounds and Derivatives and the Efficiency to Produce Hydrogen Peroxide and 5-Hydroxymethylfurfural Tolerant Cellulases

**DOI:** 10.3390/jof7100785

**Published:** 2021-09-22

**Authors:** Kakoli Chanda, Atifa Begum Mozumder, Ringhoilal Chorei, Ridip Kumar Gogoi, Himanshu Kishore Prasad

**Affiliations:** Department of Life Science and Bioinformatics, Assam University, Silchar 788011, India; kakolimic@gmail.com (K.C.); atifamozumder@gmail.com (A.B.M.); choreiringhoilal@gmail.com (R.C.); ridip.gogoi09@gmail.com (R.K.G.)

**Keywords:** lignocellulolytic endophyte, pretreatment inhibitors, tolerance, H_2_O_2_, HMF, β-glucosidase enhancement

## Abstract

Fungal endophytes are an emerging source of novel traits and biomolecules suitable for lignocellulosic biomass treatment. This work documents the toxicity tolerance of *Colletotrichum* sp. OH toward various lignocellulosic pretreatment-derived inhibitors. The effects of aldehydes (vanillin, *p*-hydroxybenzaldehyde, furfural, 5-hydroxymethylfurfural; HMF), acids (gallic, formic, levulinic, and *p*-hydroxybenzoic acid), phenolics (hydroquinone, *p*-coumaric acid), and two pretreatment chemicals (hydrogen peroxide and ionic liquid), on the mycelium growth, biomass accumulation, and lignocellulolytic enzyme activities, were tested. The reported *Colletotrichum* sp. OH was naturally tolerant to high concentrations of single inhibitors like HMF (IC_50_; 17.5 mM), levulinic acid (IC_50_; 29.7 mM), hydroquinone (IC_50_; 10.76 mM), and H_2_O_2_ (IC_50_; 50 mM). The lignocellulolytic enzymes displayed a wide range of single and mixed inhibitor tolerance profiles. The enzymes β-glucosidase and endoglucanase showed H_2_O_2_- and HMF-dependent activity enhancements. The enzyme β-glucosidase activity was 34% higher in 75 mM and retained 20% activity in 125 mM H_2_O_2_. Further, β-glucosidase activity increased to 24 and 32% in the presence of 17.76 and 8.8 mM HMF. This research suggests that the *Colletotrichum* sp. OH, or its enzymes, can be used to pretreat plant biomass, hydrolyze it, and remove inhibitory by-products.

## 1. Introduction

Lignocellulosic biomass includes any organic material, except fossil fuels, capable of being utilized as a fuel or converted into energy [1]. In the event of a scarcity of non-renewable resources, lignocellulosic biomass is considered an alternate renewable resource available at an affordable price for biofuel production [2]. Biomass feedstocks such as agricultural residue, forest and agro-industrial residues, and municipal solid waste are low cost and sustainable for biorefinery industries [3]. Lignocellulose is a heterogeneous structural biopolymer built of polysaccharides, lignin, and proteins. The polysaccharides are polymers of monosaccharides forming cellulose (40–50%), hemicellulose (25–30%), and pectin. Phenolic polymer consisting of phenylpropanoid units makes up the integral lignin part (15–20%) [4,5,6]. Waxes, vascular bundles, microfibrils, and the degree of lignification protect the polysaccharides from degradation [4]. Furthermore, the physio-chemical properties of cellulose, including crystallinity, strong interchain hydrogen bonding, weak inter-sheet hydrophobic interactions, degree of polymerization, and the presence of amorphous cellulose, make plant biomass recalcitrant to breakdown [4]. The low-cost production of biofuels from recalcitrant plant biomass depends on the efficient and inter-linked steps of pretreatment, the release of sugars from polysaccharides, and, finally, fermentation to yield fuels.

Pretreatment breakdown of the densely interwoven matrix of physical and chemical barriers in resistant lignocellulose precedes the saccharification step. Lignin, hemicellulose, crystalline cellulose, and its polymerization degree are essentially reduced to prepare the biomass for further hydrolysis [7]. Lignocellulose can be treated with various chemical, physical, and physicochemical pretreatments, which result in varying degrees of digestibility [8,9]. Monosaccharides form the primary output of the pretreatment step; however, uncontrolled chemical reactions generate secondary lignocellulose-derived molecules that inhibit saccharification and microbial fermentation. The plant biomass and pretreatment method determine the type of chemical inhibitor. The most common inhibitors are different acids, furan aldehydes, and phenolic and non-phenolic aromatic compounds [10]. Formation of furan aldehydes (2-furaldehyde or furfural and 5-hydroxymethylfurfural or HMF) in hydrolysate is by acidic dehydration of hexoses and pentose monosaccharides. HMF produces formic and levulinic acids upon further dehydration [10,11]. Hemicellulose-derived inhibitors include acetic acid, furan aldehydes, aliphatic acids, acrylic acids, and other carboxylic acids [9,12,13]. The primary lignin breakdown products are phenols, acidic and aldehydic phenolics, and various non-phenolic aromatic molecules; examples include 4-hydroxybenzoic acid, 4-hydroxybenzaldehyde, vanillin, syringaldehyde, coniferyl aldehyde, hydroquinone, gallic acid, ferulic acid, and *p*-coumaric acid [9,12,14]. Lignin and phenolic molecules are potent inhibitors of cellulases either by their direct binding and precipitation of enzymes or by adsorption on cellulose [12,14]. Lignocellulose-derived chemicals affect the fermentation step by inhibiting microbial growth and physiology. They act by inducing membrane disruption, inhibiting biochemical pathways, DNA mutations, intracellular pH and ion imbalance, ATP and amino acid metabolism alterations, and increasing intracellular oxidative stress [15,16].

Moreover, green pretreatment compounds like alkaline hydrogen peroxide (AHP) [17] and ionic liquids (ILs) [18] also affect microbes and enzymes [17,19]. Biological detoxification refers to the employment of biological agents such as natural or engineered enzymes and microorganisms to reduce the toxic impurities originating from the pretreatment step in the fermentation medium [15,20,21,22]. Biological detoxification is an economical and ecologically friendly method well integrated with bioprocesses, such as separate hydrolysis and fermentation (SHF), simultaneous saccharification and fermentation (SSF), and consolidated bioprocessing (CBP) [23,24]. Tolerance and detoxification of harmful pretreatment-derived inhibitors using microorganisms such as bacteria, filamentous fungus, and yeast are well researched and documented [11,16,25]. Among them, filamentous fungi are considered as a robust platform in lignocellulosic bioprocesses because of several adaptations such as high and stable secretion of tolerant enzymes [12], enhanced production of multiple lignocellulolytic enzymes using cheap carbon sources [26,27], and seamless integration into SHF, SSF, and CB processes [28,29].

However, new and novel strains for biorefinery interventions are desirable from abundant fungal biodiversity prevailing in unexplored niches [30]. For plant biomass-based processes, fungal strains from natural or industrial settings preferably exhibit characteristics such as (i) wide-ranging single and multiple lignocellulose-derived inhibitor tolerance [31], (ii) single source of a diverse set of enzymes for lignin and polysaccharide depolymerization [26], (iii) tolerance and stability of the enzyme repertoire toward the end products, physio-chemical parameters, and lignocellulose-derived inhibitors [16,21], (iv) amenable to microbial co-culture and consortium [32,33,34], and (v) genetically tractable for engineering [34,35].

Endophytic fungi are the most dominant microbiota of plants, existing as a symbiotic partner inside healthy plant tissues. The diversity of endophytic fungi, based on sequence-based approaches, is proposed to be between 34 and 77 million species, up from an estimated 1 million [36,37]. The phylum Ascomycota is the most domain fungal endophyte reported. In a tropical climate, dominant endophytic fungi groups include *Colletotrichum*, *Phyllosticta*, *Pestalotiopsi,* and *Xylariaceace* [36]. Researchers have investigated how endophytic fungi can be used to discover drugs, perform bioremediation, and improve plant and human health [38,39,40]. While they are less investigated for plant biomass degradation and their associated phenotypes, they have a promising role in bioprocesses and biorefinery because they have adapted to decompose plant biomass due to their lifecycle [41]. A variety of plant biomass depolymerizing enzymes are encoded in their genomes and they are naturally tolerant of various physiological stresses [41]. Endophytic fungi with enzymes for cellulose, hemicellulose, pectin, and lignin degradation were reported from a few woody [42] and non-woody plants [43], underpinning their untapped potential. However, studies on the tolerance profile of endophytic fungi or their enzymes against chemical pretreatment by-products are still in their infancy [41].

In India, *Ocimum sanctum* has long been known for its therapeutic and pharmacological uses due to its bioactive components [44]. There is a documented diversity of culturable fungi in *Ocimum* plants which has been tested for bioactivity [45]. However, a research gap exists in plant biomass deconstruction, inhibitor reduction, and biorefinery use of the reported fungal collections. The present study aims to assess the potential of the *Ocimum* fungal strain for lignocellulolytic enzyme secretion along with multiple inhibitor tolerance of both the strain and its secreted enzymes.

In this line, this study reports an endophytic fungus, *Colletotrichum* sp. strain OH, as a versatile enzyme producer for depolymerizing lignin, cellulose, hemicellulose, and pectin. Here, we investigated the tolerance of the strain OH toward representative lignocellulose-derived inhibitors; aldehydes (furfural, 5-Hydroxymethyl furfural, vanillin, *p*-hydroxybenzaldehyde), acids (levulinic, formic, gallic, and *p*-hydroxybenzoic acid), and phenolics (hydroquinone and *p*-coumaric acid). Furthermore, we tested the combinatorial effects of mixed HMF, levulinic acid, hydroquinone, and H_2_O_2_ inhibitors on the growth of the strain. The tolerance profiles of endoglucanase (1,4-β-D-glucan-4-glucanohydrolase; EC 3.2.1.4), β-glucosidase (β-D glucosidases; EC 3.2.1.21), endoxylanase (endo-1,3-beta- xylanase; EC 3.2.1.32), laccase (EC 1.10.3.2), lignin peroxidase (EC 1.11.1.13), and manganese peroxidase (EC 1.11.1.13), in the presence of single and multiple inhibitory chemicals, are reported. The reported strain is an extremophile that is tolerant to lignocellulose-derived inhibitors and physiological stresses. Moreover, the fungus was a robust secretor of inhibitor-tolerant lignocellulolytic enzymes. Remarkably, we report the stimulation of endoglucanase activity in the presence of H_2_O_2_ (25 mM) and the stimulation of β-glucosidase activities in HMF (8.8 and 17.76 mM) and H_2_O_2_ (25, 50 and 75 mM). Thus, the endophytic fungal strain reported here presents a potential candidate for applications in biorefinery processes, such as plant biomass pretreatment, lignocellulose-derived inhibitor detoxification, and enzymatic hydrolysis.

## 2. Materials and Methods

### 2.1. Isolation and Molecular Identification of Ocimum Endophyte

In this study, the fungus strain OH was isolated from *Ocimum sanctum* leaves collected from Hailakandi (Assam), India. For isolation, about 2–3 cm of the leaf segments were surface sterilized by sequentially dipping into 0.5% sodium hypochlorite (2 min) followed by 70% ethanol (2 min) and rinsed with sterile water [46]. The sterilized samples were imprinted onto potato dextrose agar media (PDA, Fluka, Buchs, Switzerland) with 100 μg/mL chloramphenicol and incubated at 30 °C until mycelium appeared.

After culturing on potato dextrose broth (PDB, HiMedia, Mumbai, India), total genomic DNA was extracted from the freshly subcultured endophytic strain OH for molecular identification by phylogenetic analysis [47]. Polymerase chain reaction (PCR) amplification of the ITS region partial 18S ribosomal RNA (rRNA) gene, ITS1, 5.8S rRNA gene, ITS2, and, separately, large subunit 28S ribosomal RNA (LSU) was performed using the primers ITS5F (5′-GGAAGTAAAAGTCGTAACAAGG-3′) and ITS4R (5′-TCCTCCGCTTATTGATATGC-3′) and LSUF (5′- ACCCGCTGAACTTAAGC-3′) and LSUR (5′- TCCTGAGGGAAACTTCG-3′) [48]. The PCR reaction (50 μL) had 50 ng of DNA template, 0.5 μM of each primer, 1 U Phusion High-fidelity DNA polymerase (Thermo Scientific), 200 µM dNTPs, and 1× HF buffer. PCR amplification was performed in a thermal cycler (Bio-Rad, Hercules, CA, USA) with the following cycling program to amplify the ITS region: initial denaturation at 98 °C for 5 min, 35 cycles at 95 °C (denaturation) for 30 s, 56 °C (annealing) for 30 s, 72 °C (extension) for 1 min, and then a final extension for 10 min at 72 °C. For amplification of LSU, cycling conditions were 98 °C for 5 min, 35 cycles of 98 °C 30 s, 52 °C for 30 s, 72 °C for 1 min, and a final extension of 72 °C 10 min. The amplified PCR products were purified using a PCR purification kit (Qiagen, New Delhi, India) and ligated into an *EcoR*V-cut (Thermo Scientific) LITMUS28 plasmid using T4-DNA ligase (Thermo Scientific, Waltham, MA, USA). The ligated products were transformed in *E. coli* TOP10F’ (Thermo Scientific). The plasmid DNA isolated from *E. coli* TOP10F’ was PEG purified and sequenced using M13 forward and reverse primers at Xcelris Labs (Ahmedabad, India). The two sequenced reads for each amplicon were edited from the chromatogram using Finch TV software (https://digitalworldbiology.com/FinchTV, accessed on 26 March 2020). A consensus contig from two reads of each amplicon was generated using the BioEdit 7.2 tool. The nucleotide sequences were deposited in the GenBank database.

The ITS and LSU sequences generated were searched for similarity by BLASTN using non-redundant (nr) and fungal type and reference material 28S rRNA/ITS nucleotide databases (http://blast.ncbi.nlm.nih.gov/, accessed on 9 July 2021) to find closely related taxa. Additionally, to obtain an approximate species-level identification, the ITS sequence was compared using BLASTN to species hypotheses (SHs) with UNITE (https://unite.ut.ee/analysis.php, accessed on 9 July 2021) [49]. For ITS sequences based on phylogenetic analysis, the corresponding sequences from TYPE strains and UNITE SH sequence hits showing 99% identity or above were used for tree building. In LSU-based phylogenetic tree constructions, the sequences used were from NCBI nr and 28S LSU databases. For multiple sequence alignment (MSA), MAFFT version 7 (https://mafft.cbrc.jp/alignment/server/, accessed on 10 July 2021) was used with default options [50]. The evolutionary history was inferred using the maximum likelihood (ML) method for ITS and neighbor-joining (NJ) for LSU sequences. The clustering of the associated taxa was tested by a bootstrap test (1000 replicates), and phylogenetic analysis was performed using MEGA X (version 10.2.6) software [51].

### 2.2. Screening of Lignocellulolytic Enzymes

The endoglucanase and xylanase activity screenings were carried out on minimal medium agar plates (in g/L, pH 5): 1.00 KH_2_PO_4_, 0.30 CaCl_2_·2H_2_O, 0.30 MgSO_4_·7H_2_O, 10.0 (NH_4_)_2_HPO_4_, 6.94 NaH_2_PO_4_·2H_2_O, 9.52 Na_2_HPO_4_·2H_2_O, and 15 agar supplemented with 1% carboxy-methylcellulose sodium salt (CMC) and birch wood xylan. Likewise, for pectinase, 1% citrus pectin was used. A fungal mycelium plug of 6 mm in diameter was placed in the center of Petri dishes, and a no inoculum plate served as a negative control. After incubation at 30 °C for 3 days, Congo red (0.1% Congo red in distilled water) was added for 15 min at room temperature as a developer until the agar surface was covered, followed by flooding the plates with 1 M sodium chloride (NaCl; SRL, Mumbai, India) for cellulase and xylanase screening [52,53]. The plates were flooded with iodine solution for 15 min at room temperature for pectinase assay [54]. The clearing zone and the colony diameters were measured to evaluate the respective enzymatic degradation indices. The degradation index (DI) was calculated using a degradation index (DI) = (CD + CZ)/CD, where CD was the colony diameter, and CZ was the clearing zone diameter.

For the screening of β-glucosidase (BGL), the fungus was grown for 5 days in liquid Mandel’s media (LMM) (g/L): 1.4 g (NH4)_2_SO_4_, 1 g KH_2_PO_4_, 0.3 g CaCl_2_, 0.3 g MgSO_4_, 0.005 g FeSO_4_, 0.016 g MnSO4·H_2_O, 0.0014 g ZnSO_4_∙7H_2_O, 0.002 g CoCl_2_, 2 mL Tween 80 [55] with 1% CMC in a 250 mL conical flask (pH 5.0, 170 rpm, 30 °C). The culture was centrifugated, and the clear supernatant (75 µL) was tested in an esculin gel diffusion assay for 5 h at 37 °C. The appearance of a dark zone was indicative of esculin hydrolysis by β-glucosidase [56].

To test the degradation ability of filter paper cellulose, the strain OH was grown in liquid mineral media (in g/L): 1.00 KH_2_PO_4_, 0.30 CaCl_2_∙2H_2_O, 0.30 MgSO_4_·7H_2_O, 10.0 (NH_4_)_2_HPO_4_, 6.94 NaH_2_PO_4_·2H_2_O, 9.52 Na_2_HPO_4_·2H_2_O containing as a carbon source one strip of Whatman #1 filter paper (Merck Millipore, Burlington, MA, USA) and a no filter paper tube was used as a control [57].

Similarly, laccase activity (Lac) was examined using minimal salt medium (MSM) consisting of (g/L) of Na_2_HPO_4_ (2.4), K_2_HPO_4_ (2.0), NH_4_NO_3_ (0.1), MgSO_4_ (0.01), CaCl_2_ (0.01), 1 mL of sterilized 20% *w*/*v* aqueous glucose supplemented with 0.1% *w*/*v* ABTS (2-2′-azino-bis (3-ethylbenzothiazoline-6-sulphonic acid). The actively growing mycelium plug was put in the sterilized medium and incubated at 30 °C in the dark. The development of a green color indicated laccase activity and the formation of the ABTS–azine complex [58].

The assay for lignin peroxidase (LiP) activity was carried out in a reaction mixture of 2.2 mL of culture crude supernatant, 0.1 mL of methylene blue, 0.3 mL 0.5 M sodium tartrate buffer (pH 4), and 0.1 mL 45 mM H_2_O_2_. A color change from blue to green was indicative of the presence of lignin peroxidase [59].

Secreted manganese peroxidase (MnP) was detected using a mixed assay of 0.1 mL of culture crude supernatant, 0.4 mM methyl catechol, 0.2 mM MnSO_4_, 50 mM sodium succinate buffer (pH 4.5), and 0.1 mM H_2_O_2_. A change in color from pale yellow to light brown/yellowish indicated the presence of MnP [60].

### 2.3. Effect of Pretreatment-Derived Inhibitors on Colletotrichum sp. OH Growth and Biomass Accumulation

The mycelium growth on the Petri plate (9 cm diameter) and dry biomass accumulation in liquid culture in the presence and absence of inhibitory chemicals were used to evaluate the tolerance toward various aldehydes (5-Hydroxymethyl furfural; HMF, furfural; FU, vanillin; VA, *p*-hydroxybenzaldehyde; PHB), acids (levulinic acid; LA, formic acid; FA, gallic acid; GA, *p*-hydroxybenzoic acid; POH), and phenolics (hydroquinone; HQ and *p*-coumaric acid; PCA), respectively. The fungus was grown in mineral medium (MM, pH 6, g/L); KH_2_PO_4_ (0.5), K_2_HPO_4_ (1.5), NH_4_Cl (3), MgSO_4_ (0.3), CaCl_2_ (0.01), FeCl_3_ (0.01). The medium was supplemented with 1% glucose and single inhibitor (sterilized by 0.22 μm syringe filters; PVDF, Millipore, Darmstadt, Germany) at different concentrations (0–30 mM). The control cultures for relative growth and biomass accumulation were without any inhibitors. The mycelium growth was recorded by measuring the colony diameters (cm) of the fungus growing on solid plates (2% agar) until the control plate inoculum covered the entire plate (7 days) at 30 °C.

The dry weight biomass was estimated by growing 1 × 10^5^ conidia in liquid MM supplemented with and without inhibitors for 7 days. Dry weight was measured for a 30 mL sample that was filtered, washed twice with distilled water, and dried at 65 °C to a constant weight. Growth inhibition was the percentage difference in relative growth/biomass between each culture (with inhibitors) and the control culture (without inhibitors) [61]. Additionally, mycelial growth in the presence of lignocellulosic-derived sugars was measured by growing strain OH on mineral media supplemented with 10% (*w*/*v*) of glucose, xylose, arabinose, and galactose.

### 2.4. Effect of Pretreatment Compounds and Cell Wall Stress Response

Mineral media supplemented with different concentrations of hydrogen peroxide (H_2_O_2_; 0–50 mM), menadione (0–100 µM), Calcofluor white (CFW, 500 µg/mL), Congo red (CR, 1000 µg/mL), and sodium dodecyl sulfate (SDS, 1%) were used to evaluate the oxidative and cell wall stress tolerance of the fungus [62]. Growth inhibition (%) in the presence and absence of stress was analyzed by measuring the relative growth and dry weight biomass described previously. The inhibitory effect of ionic liquid (IL), 1-butyl-3-methylimidazolium chloride (Bmim Cl) with respective control NaCl (2% and 5%) on OH growth and biomass accumulation was measured similarly.

### 2.5. Effects of Different Combinations of Inhibitor on Colletotrichum sp. OH Growth and Biomass Accumulation

A manual model was developed for combinations, and the experimental plan had 16 trials with control (Table 1). To test the tolerance of the combinatorial treatments, we initially calculated the inhibitory threshold concentration values by estimating the concentration at which the growth was reduced by 50% (IC_50_) by estimating mycelium growth at 7 days of cultivation from the abovementioned experiments (Section 2.3 and Section 2.4). The IC_50_ values were calculated by generating an inhibitor vs. dose–response curve for each chemical inhibitor tested using the software GraphPad Prism 5 software version 5.01. The multiple chemical inhibitor tolerance experiments were conducted using four inhibitors, HMF, levulinic acid, hydroquinone, and H_2_O_2_, with IC_50_ values of 17.76 mM, 29.77 mM, 10.76 mM, and 50 mM, respectively (Section 3.2).

### 2.6. Lignocellulolytic Enzymes Production in Shaking Flasks

For lignocellulolytic enzyme production, strain OH was grown in 500 mL flasks containing 50 mL LMM (pH 6) with the following plant biomass as a carbon source (1% *w*/*v*): CMC, Avicel^®^ PH-101 (Av), oil cake (OC, obtained when mustard oil was extracted from mustard seed kernels and dried), rice husk (Rh), and glucose (Gl). Likewise, 1% of rice husk for ligninase and 1% of citrus pectin for pectinase as the sole carbon source were used for the culture conditions. The culture media were inoculated with 1 × 10^5^ conidia/mL and incubated on a shaking incubator (Orbitek, Chennai, India) at 30 °C, 170 rpm for 7 days. The cell-free supernatant was harvested by centrifugation (Eppendorf 5804R) at 10,000 rpm for 10 min at 4 °C and used as a crude enzyme to measure lignocellulolytic enzyme activity.

### 2.7. Enzyme Assays

Total cellulase (FPase) and endoglucanase activity were estimated using Whatman filter paper No. 1 (50 mg) and CMC (2% *w*/*v*) as the substrate in sodium citrate buffer (50 mM, pH 5) following the protocol of Ghose [63]. The exoglucanase activity was determined using 4% (*w*/*v*) Avicel as the substrate in sodium citrate buffer (50 mM, pH 5) following the protocol of Rai et al. [64]. Xylanase assay was performed by following the protocol described by Bailey et al. [65] using 1% birch wood xylan in sodium citrate buffer (50 mM, pH 5). For each assay, a substrate blank and enzyme blank were also run alongside. The amount of releasing sugars was measured following the protocol of Miller [66] using dinitrosalicylic acid (DNS) reagent at 540 nm in a spectrophotometer (Lambda 25 UV/VIS, PerkinElmer, Waltham, MA, USA). One unit of endoglucanase, exoglucanase, FPase, and endoxylanase activity was defined as the amount of enzyme that released 1 μmol of reducing sugar (glucose/xylose) per minute under the given assay conditions. The β-glucosidase activity was assayed using *p*-nitrophenyl-β-D-glucopyranoside (*p*NPG) as the substrate prepared in sodium citrate buffer (50 mM, pH 5). The reaction mixture was incubated at 50 °C for 10 min in dark conditions. The reaction was terminated by adding 1 M sodium carbonate, and the absorbance of *p*-nitrophenol released was measured according to Gao et al. [67]. One unit of β-glucosidase activity was defined as the amount of enzyme that released 1 μmol of *p*-nitrophenol per minute under the assay conditions at 405 nm using a spectrophotometer.

Similarly, pectinase activity was quantitatively measured using 0.5% citrus pectin in sodium citrate buffer (50 mM, pH 5) [68]. Enzyme activity was defined as the amount of enzyme required to generate 1 µmol of galacturonic acid per min under the assay conditions at 540 nm. The activities were calculated using the following equation: enzyme activity (U/mL) = µg galacturonic acid released × *V*/*v* × 194.1 × *t* (1), where *V* was the total volume of solution, *v* was the volume of the crude enzyme used in the assay, 194.1 was the molecular weight of galacturonic acid, and *t* was the reaction time in min.

The lignin peroxidase activity was measured by H_2_O_2_-dependent oxidation of veratryl alcohol. The reaction mixture contained 500 µL of culture filtrate, 500 µL 10 mM veratryl alcohol, and 1 mL of 125 mM sodium tartrate buffer (pH 3). The reaction was started by adding 500 µL of 2 mM H_2_O_2_ and incubating at 30 °C for 5 min. The change in absorbance was continuously monitored using a spectrophotometer at 310 nm (€= 9300 M/cm). Enzyme activity was defined as one unit of enzyme activity for the oxidation of 1 µmol of veratraldehyde (oxidized product of veratryl alcohol) produced per min per mL of culture filtrate [69]. Laccase activity was determined by the oxidation of ABTS to ABTS–azine. The assay reaction mixture contained 0.5 mM of ABTS, 2.8 mL of 0.1 mM sodium acetate buffer (pH 4.5), and 100 μL of culture supernatant. After incubating for 5 min at 30 °C, the absorbance was read at 420 nm (€ = 36,000 M/cm) using a spectrophotometer. Enzyme activity was defined as one unit of laccase involved in the oxidation of 1 μmol of ABTS substrate per min [70]. Manganese peroxidase activity was estimated by oxidation of 2,6-Dimethoxyphenol (2,6-DMP) to 3,3′,5,5′-tetramethoxy-p, p′-diphenoquinone in the presence and absence of Mn^2+^. The reaction mixture consisted of 1 mM 2,6-DMP, 0.1 mM H_2_O_2_, 1 mM MnSO_4_, and 100 mM sodium tartarate (pH 4.5). The oxidation of 2,6-DMP was estimated by reading absorbance at 469 nm (€ = 6500 M/cm). One unit of manganese peroxidase activity was defined as 1 µmol of DMP oxidized per min under the assay conditions [71].

The Lac, LiP, and MnP activity was calculated using the following formula: enzymatic activity (U/mL) = (A * *V*)/(*t* * € * *v*), where A = absorbance (nm), *V* = total volume of reaction mixture (mL), *v* = supernatant volume (mL), *t* = incubation time (min), and € = extinction coefficient (M/cm).

### 2.8. Native Poly Acrylamide Gel Electrophoresis (PAGE) and Zymography

The total protein concentration was determined according to Bradford [72] using bovine albumin serum (Affymetrix, Santa Clara, CA, USA) prepared in citrate buffer (50 mM, pH 5) as a standard with respective control (culture supernatant replaced by buffer). PAGE was conducted using 10% native gel containing CMC and xylan (1% *w*/*v*). The well in the respective gel was loaded with an equal amount of diluted 6th day culture supernatant (protein concentration; 10 µg/µL) from respective culture conditions as discussed in Section 2.7 and run at 4 °C, 90 V for 4 h using an electrophoresis system (Sub-cell^®^ GT Agarose Gel; Bio-Rad). After the run, the gel with protein marker (Precision Plus Protein^TM^ Standards; Bio-Rad) was stained with Coomassie brilliant blue R-250 (Affymetrix) while the CMC and birch wood xylan incorporated gel was incubated at 50 °C for 30 min in 50 mM sodium citrate buffer (pH 5). After incubation, the gel was transferred to a 0.1% Congo red solution for 20 min and de-stained using 1 M NaCl to visualize the distinct hydrolysis zone. Exoglucanase and BGL were visualized by soaking the gel in 5 μM of 4-methylumbelliferyl-β-D-cellobioside (MUC) and 4-methylumbelliferyl- β-D-glucopyranoside (MUG) in sodium citrate buffer (pH 5) [73], and the fluorescence signals were captured using a UV transilluminator in ChemiDoc^TM^ MP (Bio-Rad).

### 2.9. Tolerance of Lignocellulolytic Enzymes in the Presence of Single and Combinations of Inhibitors

A plate-based assay was used to test the effect of chemical inhibitors on enzyme production and tolerance. The fungus was grown on 1% CMC plates amended with different combinations (Table 1) of inhibitors at their IC_50_ concentration and grown for 7 days. The secreted endoglucanase (CMCase) forms a halo zone, indicating CMC degradation and tolerance (Section 2.2).

Lignocellulosic enzymes secreted by the fungus were tested for their tolerance to 16 combinations using crude supernatant. For this, the fungus was grown in a 2 L flask in a 500 mL production medium (LMM) with 1% rice husk (Rh) as a carbon source for 6 days, 170 rpm, 30 °C (Section 2.6). A flask with no inoculum was used as a control. A total of 500 mL crude culture supernatant was filtered through a 0.25 mm membrane (PVDF), then concentrated using Viva Flow 250 (10 kDa cutoff) (Sartorius, Gottingen, Germany) at 4 °C. Then, the concentrated filtrate was suitably diluted in respective enzyme-specific buffer, and an equal amount of concentrated filtrate (1 mg) was subsequently used for the various lignocellulolytic activities. The effect of different combinations of inhibitory chemicals as described in Table 1 was evaluated at their IC_25_, IC_50_, and IC_75_ values (mM). The enzyme activity was evaluated by incubating the crude concentrated bulk extract with the respective enzyme-specific substrate supplemented with 16 combinations of inhibitors following the enzyme assay protocol discussed in Section 2.7. Likewise, control assays were performed without any inhibitors. All experiments were analyzed in terms of the percentage of relative activities (%) concerning the control assay (without inhibitors).

### 2.10. Effect of Exogenous H_2_O_2_ on β-Glucosidase Activity

The effect of H_2_O_2_ (25, 50, 75, 100, and 125 mM) on BGL activity was analyzed by measuring the relative activity. The reaction mixture consisted of 5 mM *p*-nitrophenyl-β-D-glucopyranoside (*p*NPG) as the substrate in 50 mM citrate buffer (pH 5) supplemented with various concentrations of H_2_O_2_ (25, 50, 75, 100, and 125 mM). The reaction mixture was incubated at 50 °C for 10 min in dark conditions. The reaction was terminated by adding 1 M sodium carbonate, and the absorbance of *p*-nitrophenol released was measured [67]. The *p*-nitrophenol released in the presence of BGL was compared to the blank assay; the buffer replaced culture supernatant and H_2_O_2_. Further, the tolerance capability of the BGL enzyme in the presence of 75 mM H_2_O_2_ was investigated by pre incubating the bulk crude extract in the buffer for 0–6 h supplemented with H_2_O_2_. The 75 mM H_2_O_2_ corresponds to the highest concentration at which the enzyme retains more than 100% of its activity. The relative enzyme activity with respect to control was measured as discussed in Section 2.9.

### 2.11. Statistical Analysis

The results are expressed as mean ± SEM from data collected across experimental repeats (*n* = 3 per data point, a biological triplicate, and repeated experiment). Data were analyzed using analysis of variance (ANOVA) with post hoc Tukey’s means as a comparison to find a significant difference. Significance was at a *p*-value < 0.05. All the statistical analyses were carried out using OriginPro 8.5 and GraphPad Prism 5 software version 5.01.

## 3. Results

### 3.1. Identification and Screening of Ocimum Endophyte Showing Potential Lignocellulose Degradation

**Figure 1:**
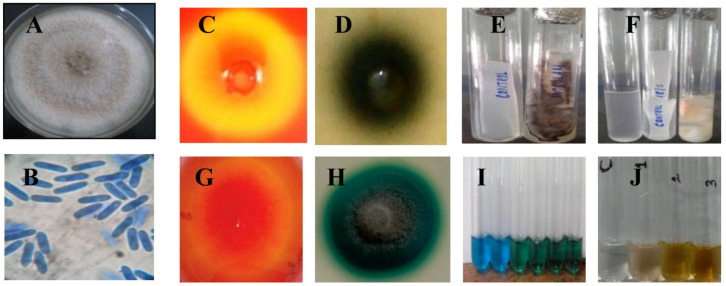
Screening for the lignocellulolytic potential of the strain OH. (**A**). Colony morphology of OH on PDA plate after 7 days at 30 °C; (**B**) microscopic appearance at magnification 100×; after 7 days at 30 °C; and (**C**–**J**) lignocellulolytic enzyme profile of OH. (**C**) Cellulose hydrolysis on CMC agar on the 3rd day of screening; (**D**) BGL plate screening using esculin as a substrate; (**E**,**F**) cellulose-degrading capabilities in minimal medium containing Whatman filter paper strip. Images after 7 days incubation. (**E**) Left tube: control without inoculum in solid media and (**F**) in liquid culture. (**G**) Screening for xylanase on a xylan agar plate. (**H**) Laccase activity was tested in an MSM containing ABTS (2-2′-azino-bis (3-ethylbenzothiazoline-6-sulphonic acid). (**I**) Lignin peroxidase assay with methylene blue, left tube (1st tube): control without culture supernatant and (**J**) manganese peroxidase assay with methyl catechol, left tube (1st tube): control without culture supernatant.

One of the *Ocimum sanctum* leaf endophytic fungal isolates (designated OH) was selected based on the preliminary cellulose degradation capacity. The fungus displayed a fast growth on potato dextrose agar and filled the entire plate (90 mm dia) in 7 days at 30 °C. Dense white filamentous mycelium with light orange and creamy conidial droplets formed near the inoculum point on maturation (Figure 1A). The microscopic observation showed simple hyaline ovoid conidia (Figure 1B). The rapid preliminary screening of the fungal strain OH for endoglucanase activity displayed a clearance zone, indicating cellulase production (Figure 1C). Further, the strain OH was screened for the production of various lignocellulolytic enzymes such as β-glucosidase (BGL), filter paper cellulase (FP), endoxylanase, and ligninase based on qualitative plate screening. A sign of BGL activity is the presence of a black zone of diffusion in esculin agar (Figure 1D). Simultaneously, for the remaining enzymes tested, the strain showed a zone of color formation in the respective solid and broth culture as depicted in Figure 1E–J.

**Figure 2:**
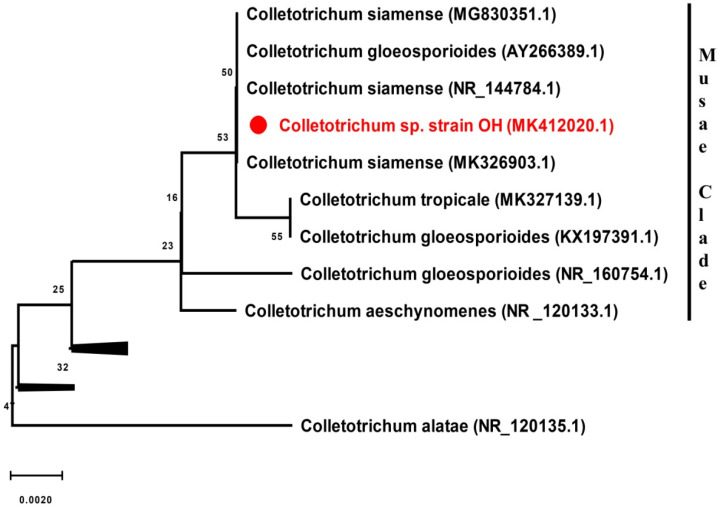
Rooted maximum likelihood phylogenetic tree using ITS gene sequences. The phylogenetic tree depicts *Colletotrichum* sp. strain OH (red filled circle and red text) with Musae clade of *Colletotrichum gloeosporioides* species complex. Accession numbers are in parentheses; Bootstrap values (based on 1000 replications) are shown at the branch points. Scale bar = 0.0020 substitutions per nucleotide position. A compressed tree is presented for clarity, and the complete tree is presented in Figure S1.

The sequence-based identification of the fungus was performed with phylogenetic trees based on the nucleotide sequences of partial SSU-ITS1-5.8S rRNA-ITS2-partial LSU (i.e., ITS) and LSU region of the rDNA. The identical sequences (99% and above) of the related species were obtained from BLASTN searches of ITS and LSU gene sequences (GenBank accession numbers MK412020.1 and MK411296.1) using default parameters. The ITS-based ML phylogenetic tree (Figure 2, Figure S1) clustered the strain OH sequence and several type isolates of *Colletotrichum siamense* and *Colletotrichum gloeosporioides.* Additionally, the ITS-based UNITE analysis identifies (at 0.0% threshold) the strain OH as *Colletotrichum* at the taxon hypothesis level (DOI:TH033307) and *Colletotrichum siamense* at the species hypothesis (SH) level. The SH (SH2218989.08FU; DOI:SH2218989.08FU) consists of 604 clustered identical ITS sequences with the majority corresponding to *Colletotrichum gloeosporioides* (168), *Colletotrichum* (65), and *Colletotrichum siamense* (45). The fungus OH was identified as *Colletotrichum* sp. strain OH based on the ITS/LSU phylogenetic analysis and available data [74,75,76,77]; it belonged to the Musae clade of the *Colletotrichum gloeosporioides* species complex (Figure 2). Similarly, the phylogenetic tree prepared with LSU sequences to identify the strain OH is presented in Figure S2. The nucleotide sequences were submitted to the GenBank database with accession numbers MK411296.1 and MK112020.1.

### 3.2. A Broad-Spectrum Tolerance Capability of Colletotrichum sp. OH to Pretreatment-Generated Inhibitory Compounds

**Figure 3:**
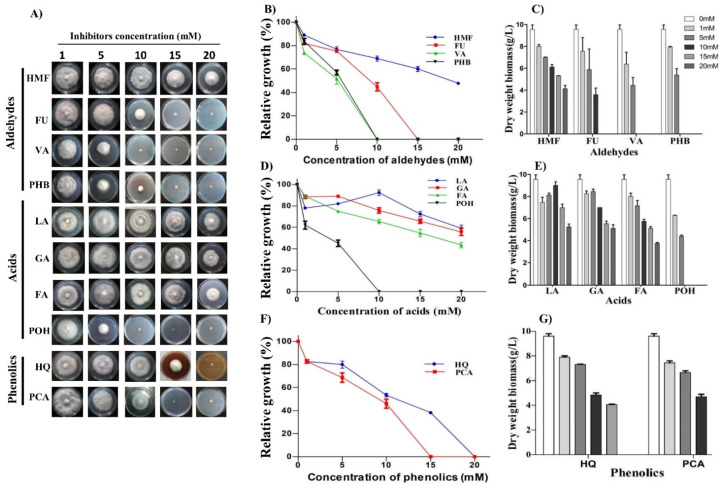
Tolerance of *Colletotrichum* sp. OH to lignocellulosic-derived inhibitors. (**A**) The mycelial growth in the presence of three groups of inhibitors; aldehydes, acids, and phenolics (HMF, FU, furfural; VA, vanillin; PHB, *p*-hydroxybenzaldehyde; LA, levulinic acid; FA, formic acid; GA, gallic acid; POH, *p*-hydroxybenzoic acid; HQ, hydroquinone; PCA, *p*-cumaric acid). (**B**,**D**,**F**) is the relative mycelial growth (%) in plates and (**C**,**E**,**G**) dry weight biomass (g/L) of OH on the seventh day when supplemented with and without inhibitors. The control (no inhibitors) plate showed 9 cm mycelial growth on the 7th day of incubation. Experiments were performed in triplicate, and the heights of the bars and the error bars show the means ± standard error mean (SEM).

We tested the tolerance limits of the strain OH toward several lignocellulosic biomass-derived inhibitors, ranging from 0–30 mM, both on solid mineral medium plates and mineral broth culture. Both mycelial growth and dry biomass were found to determine the concentration and inhibition threshold of these chemicals. The tolerance on solid plates is reported as a growth percentage (%) with regard to no inhibitor plates, while biomass is reported as g/L. The plate screenings for determining the tolerance of the OH to different groups of inhibitors are shown in Figure 3A. Further, IC_50_ values were calculated for each inhibitor by preparing an inhibitory threshold curve in GraphPad Prism software using mycelium diameter. Thus, the concentration (IC_50_) of each inhibitory compound that inhibited *Colletotrichum* sp. OH growth by 50% was determined. The data suggested that the threshold concentration varied from inhibitor to inhibitor and even within the same aldehydic, acidic, and phenolic groups (Table 2).

The four primary lignocellulose-derived inhibitors, 5-hydroxymethylfurfural (HMF) and furfural (furan derivatives) and vanillin and *p*-hydroxybenzaldehyde (aromatic aldehydes), were tested in the range of 0–20 mM. Among the tested aldehydes, strain OH showed maximum tolerance to HMF compared to growth on furfural and the two other aromatic aldehydes. Growth inhibition by vanillin and *p*-hydroxybenzaldehyde had a similar profile. At 5 mM of vanillin and *p*-hydroxybenzaldehyde, the relative growth and biomass measured were 52 ± 0.32%, 57 ± 0.26%, 4.43 ± 0.12 g/L, and 5.38 ± 0.43 g/L, respectively. At concentrations of 10 mM and above, both aromatic aldehydes resulted in drastic growth reduction. In contrast, furan aldehydes HMF and furfural showed weaker inhibition at a concentration of 5 mM, resulting in 77 ± 0.16% and 76 ± 0.04% relative growth. Depending on the inhibitor, increasing the concentration of furan aldehydes resulted in a progressive reduction in relative growth and biomass (Figure 3B,C). On 10 mM furfural-amended media, relative growth dropped to 44 ± 0.11% (Figure 3B). The growth and biomass were negligible on media with 15 and 20 mM furfural. In contrast, relative growth and biomass were 59.2 ± 0.16 and 48 ± 0.07% on 15 and 20 mM HMF, suggesting tolerance to HMF (Figure 3B,C).

We systematically investigated the tolerance potential of the fungus to four different acids: gallic, formic, levulinic, and *p*-hydroxybenzoic acid (range 0 to 30 mM) (Figure 3D,E). Among the tested acids, *p*-hydroxybenzoic acid showed the most potent inhibitory effect beyond a 5 mM concentration. The inhibitory potential calculated as IC_50_ was of the order: *p*-hydroxybenzoic acid (4.94 mM) > formic acid (15.87 mM) > gallic acid (25.87 mM) > levulinic acid (29.77 mM). The relative growth and biomass of OH were about 62 ± 0.19%, 45 ± 0.08%, 6.3 ± 0.04 g/L, and 4.3 ± 0.13 g/L, respectively, in the presence of 1 mM and 5 mM of *p*-hydroxybenzoic acid (Figure 3D,E).

The relative growth and biomass accumulation were not affected much by formic acid (up to 15 mM). In comparison, gallic and levulinic acid caused less inhibition when up to 20 mM with relative growth and biomass of 56 ± 0.08%, 59 ± 0.09%, 5.16 ± 0.20 g/L, and 5.4 ± 0.13 g/L, respectively. Levulinic acid exerted the most negligible impact on the relative growth and biomass of OH. The biomass yield and relative mycelium growth were reduced by approximately 44 ± 0.09% and 5.43 ± 0.1 g/L at 20 mM of levulinic acid. The inhibitory effects of formic acid on OH had a similar trend to levulinic acid. At a 15 mM formic acid concentration, the relative growth and biomass contents were 55 ± 0.16% and 5.2 ± 0.18 g/L, respectively. Thus, the strain OH can tolerate levulinic acid more than any other acids tested. Further, we tested the effect of two exogenous phenolics, hydroquinone (HQ) and *p*-coumaric acid (PCA) (0–20 mM each), prominent lignocellulolytic pretreatment by-products, on the growth of the fungus. The IC_50_ values calculated were 10.76 mM for hydroquinone and 8.63 mM for *p*-coumaric acid (Figure 3F,G). The relative growth and biomass of OH were 50 ± 0.24% and 5 ± 0.15 g/L, respectively, on 10 mM hydroquinone. However, *p*-coumaric acid completely blocked the mycelial growth above 10 mM (Figure 3F,G).

Among the inhibitors, the highest IC_50_ was for LA (29.77 mM), HMF (17.76 mM), and HQ (10.76 mM) (Table 2), suggesting that the fungus *Colletotrichum* sp. OH can tolerate them (Figure 3A–C). Additionally, the strain OH showed growth on several lignocellulosic-derived sugars at a concentration of 10% (Figure S3). Among the sugars, the maximum tolerance was glucose, followed by xylose, galactose, and arabinose.

### 3.3. Wide-Ranging pH, Oxidative, and Cell Wall Stress Tolerance in Colletotrichum sp. OH

**Figure 4:**
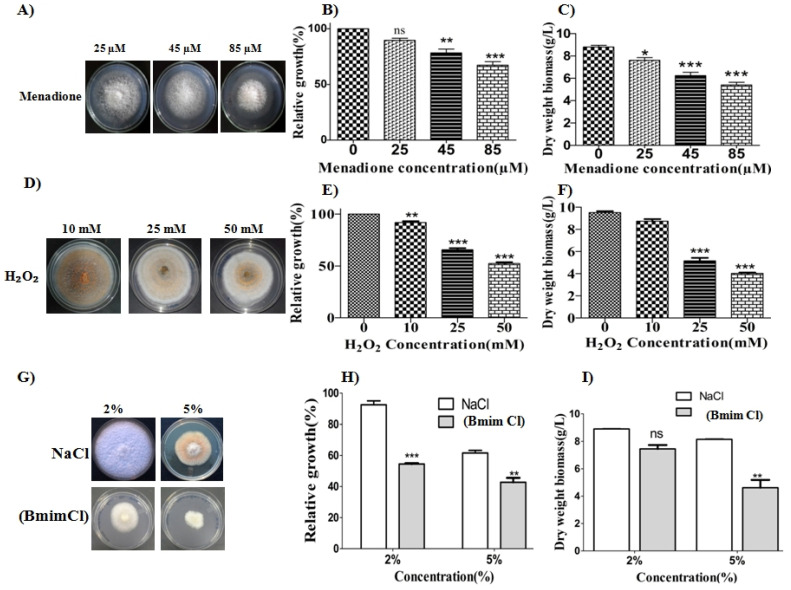
Effect of lignocellulosic pretreatment compounds on *Colletotrichum* sp. OH growth. Oxidative stress response of OH to menadione and H_2_O_2_. (**A**,**D**) The mycelial growth in the presence of menadione and H_2_O_2_; (**B**,**C**,**E**,**F**) the relative mycelial growth (%) and dry weight biomass (g/L) of OH on the seventh day; and (**G**–**I**) the relative growth (%) and dry weight biomass (g/L) in the presence of ionic liquid 1-butyl-3-methylimidazolium chloride (Bmim Cl). Statistical differences were analyzed by one-way analysis of variance (ANOVA). Experiments were performed in triplicate, and the heights of the bars and the error bars indicate the means ±standard error mean (SEM). *** *p* < 0.001; ** *p* < 0.01; * *p* < 0.05 compared with the control.

We further tested the pH tolerance of OH by growth measurements on solid and liquid media. The fungus showed a wide range of pH tolerance (4–13) with maximum growth at pH 6 (Figure S4). Next, the tolerance of the strain OH toward two oxidant species, hydrogen peroxide (H_2_O_2_) and menadione, was tested. Although the two compounds impart oxidative stress, the results showed distinct responses, with menadione showing an inhibitory effect above 85 µM as shown in Figure 4A–C. H_2_O_2_, on the other hand, showed negligible inhibition at 10 mM, and a concomitant reduction in growth and biomass accumulation occurred at higher concentrations. Interestingly, higher concentrations of H_2_O_2_ significantly altered the colony morphology of the strain OH (Figure 4D). Thus, OH has a remarkably higher tolerance to H_2_O_2_ as the relative growth was reduced to 50% when the concentration reached 50 mM (Table 2).

Among the cell wall stress inducers (Congo red, sodium dodecyl sulfate, and Calcofluor) OH showed significantly higher tolerance to Congo red, reaching a concentration up to 1 mg/mL (Figure S5). In addition, the fungus produced a clear halo around the colony, demonstrating its dye decolorization and degradation capability. The fungus could also tolerate CFC and SDS at high concentrations, suggesting its robust cell wall integrity mechanisms (Figure S5).

### 3.4. The Toxicity Tolerance of Colletotrichum sp. OH to Ionic Liquid

The use of ionic liquids (ILs) is a green alternative for pretreating lignocellulose materials. ILs, such as 1-butyl-3-methylimidazolium chloride (Bmim Cl), can be efficiently used to pretreat lignocellulosic biomass, and we further investigated the effects of IL on the growth of the fungus. In all cases, a drop in radial growth was noticed with increasing concentrations of IL (Figure 4G–I). It was noteworthy that even at a high concentration of 5% (*w*/*v*), the strain could grow, but the relative growth was reduced by more than half compared to control NaCl.

### 3.5. Tolerance of Inhibitor Combinations by Colletotrichum sp. OH

In this study, we further tested the potential of the fungus to tolerate different combinations of inhibitors. For this, we used HMF, levulinic acid, hydroquinone, and H_2_O_2_, at their sublethal half-maximal inhibitory concentration (IC_50_; mM, Table 2) values to assess the tolerance to multiple inhibitors simultaneously. Here, we created a manual model of a total of 16 combinations (Table 1), including no inhibitor (combination 1) and single, two, three, and all four inhibitors (combinations 2–16) at their IC_50_ concentrations as determined by individual inhibitor growth profiles. The growth inhibition of the strain OH was examined on the solid and liquid MM cultures and represented as relative mycelial growth and biomass values compared to no inhibitory control (Table 3). As expected, the growth inhibition increased with an increase in inhibitor supplementation. For the six dual combined inhibitors, the relative biomass decreases recorded were 61.1% for combination 6 (17.76 mM HMF, 10.76 mM HQ); 56.5% for combination 7 (17.76 mM HMF, 50 mM H_2_O_2_); 54.7% for combination 8 (17.76 mM HMF, 29.77 mM LA); 53.3% for combination 12 (29.77 mM LA, 50 mM H_2_O_2_); 58% for combination 13 (29.77 mM LA, 10.76 mM HQ); and 54.3% for combination 15 (10.76 mM HQ, 50 mM H_2_O_2_). Interestingly, the dual combinations exerted less severe inhibitions, and the additive effects of growth inhibition were negligible. Remarkably, the dual combinations where H_2_O_2_ was a partner showed marginally better or lower biomass inhibition than H_2_O_2_ alone. The biomass inhibition decreased by 2.62 and 0.73% in combinations 12 (H_2_O_2_ + LA) and 15 (H_2_O_2_ + HQ), while marginally increasing by 3.18% in combination 7 (H_2_O_2_ + HMF) and combination 5 (H_2_O_2_). The highest increase in biomass inhibition was 40% in combination 13 (LA + HQ) and combination 3 (LA). The dual combination of HMF with levulinic acid, i.e., combination 8 (17.76 + 29.77 mM), showed only 38.9% relative mycelial growth inhibition, suggesting it was the best tolerated combination on the solid medium. Further, we tested four triple combinations for tolerance testing. The relative biomass inhibition was 71.0% for combination 9 (17.76 mM HMF, 29.77 mM LA, 50 mM H_2_O_2_); 69.2% for combination 10 (17.76 mM HMF, 29.77 mM LA, 10.76 mM HQ); 77.5% for combination 11(17.76 mM HMF, 10.76 mM HQ, 50 mM H_2_O_2_); and 71% for combination 14 (29.77 mM LA, 10.76 mM HQ and 50 mM H_2_O_2_). When comparing the common dual and triple inhibitor combinations, the lowest increase in biomass inhibition was 11.7% for combination 10 (HMF + HQ + LA) compared with combination 6 (HMF + HQ). The highest biomass inhibition increase was 29.93% for combinations 11 (HMF + HQ + H_2_O_2_) and 15 (HQ + H_2_O_2_). The biomass germination failed in the presence of four inhibitors, combination 16 (17.76 mM HMF, 29.77 mM LA, 10.76 mM HQ, and 50 mM H_2_O_2_), while the solid plate mycelium growth showed 77% inhibition. The biomass inhibition increase was lowest (22.5%) for combination 16, compared with combination 11 (HMF + HQ + H_2_O_2_), and highest (30.8%) with combination 10 (HMF + LA+ HQ).

### 3.6. Lignocellulolytic Enzyme Production and Secretome Analysis

**Figure 5:**
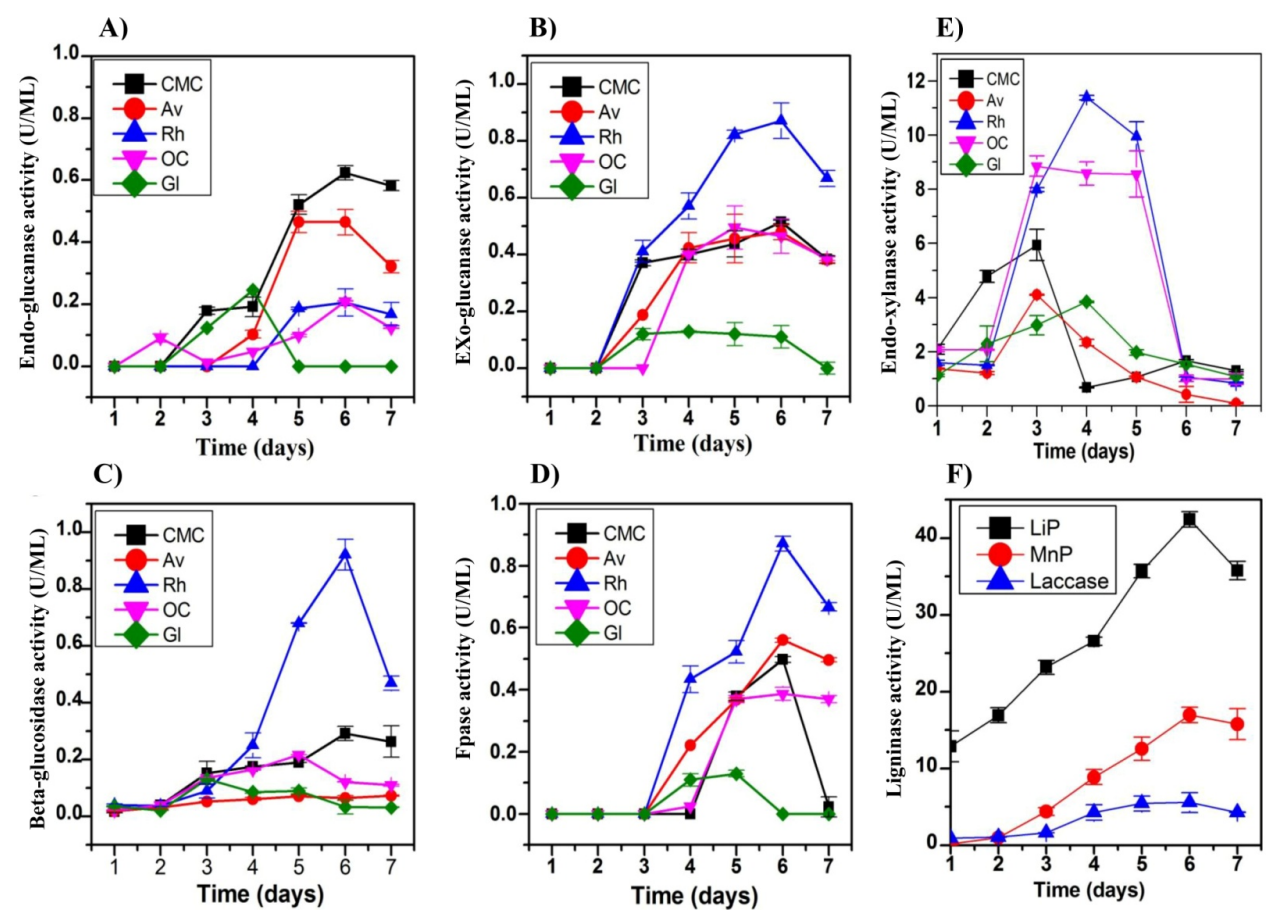
Activities of extracellular lignocellulolytic enzymes of *Colletotrichum* sp. OH, in the presence of different carbon sources. Time-series plots of endoglucanase (**A**), exoglucanase (**B**), β-glucosidase (**C**), Fpase (**D**), and endoxylanase (**E**) production by OH on different carbon sources. (**F**) Changes in ligninase activities, such as Lac, laccase; MnP, manganese peroxidase; and Lip, lignin peroxidase, in the presence of rice husk as detected by the colorimetric assay. Experiments were performed in triplicate, and the heights of the bars and the error bars indicate the means ± standard error mean (SEM).

The strain OH showed rapid growth on solid plates amended with rice husk and oil cake, suggesting the potential to utilize plant biomass (Figure S6). Next, we tested the production of multiple plant biomass-degrading enzymes using 1% carboxymethyl-cellulose, Avicel, rice husk, oil cake, and glucose as the sole carbon source. The bulk secretome from the submerged fermentation was assayed for six different enzymes for seven days (Figure 5A–F). The endoglucanase activity increased with the incubation time (Figure 5A), and the maximum activity (0.62 ± 0.053 U/mL) was from the CMC culture secretome on the 6th day. The exoglucanase activities under different carbon sources (Figure 5B) showed a similar increase around the 3rd day and a gradual peak around the 6th day. The maximum exoglucanase and FPase activities were 0.871 ± 0.2 U/mL and 0.56 ± 0.2 U/mL, respectively, from the rice husk secretome. The highest BGL activity was for oil cake and CMC (8.4 ± 0.19, 8.2 ± 0.45 U/mL) secretomes. (Figure 5D). No cellulase activity was detected in the glucose-grown culture. The xylanase activity showed a gradual increase in the presence of rice husk that reached 11.49 ± 0.176 U/mL in the 4th day culture. Xylanase activity was detected in the glucose- and Avicel-treated culture, with the highest activity of 8.32 ± 0.22 U/mL on the 5th day in the oil cake secretome (Figure 5E). Likewise, the fungal strain showed a measurable amount of pectinase production in 1% citrus pectin induction culture (Figure S7).

The fungus secretome was evaluated for the lignin-degrading enzymes (e.g., Laccase; Lac, Manganese peroxidase; MnP, and Lignin peroxidase; LiP) from activities in the rice husk-grown culture (Figure 5F). The strain OH showed the production of all three enzymes with maximum activities recorded on the 6th day. The peak production of lignin peroxidase was 41.22 ± 0.03 U/mL on the 6th day, after which the activity decreased. However, the activity (U/mL) of MnP and laccase enzyme production varied with LiP production.

**Figure 6:**
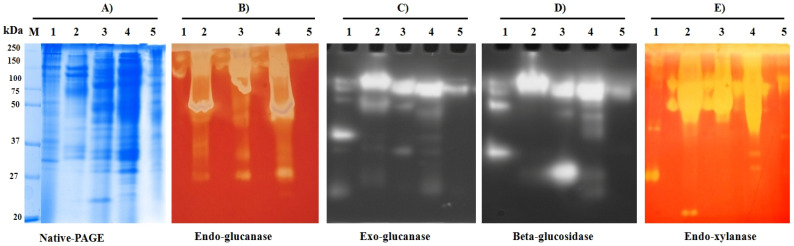
Native PAGE and zymogram analysis of cellulases and xylanase produced by *Colletotrichum* sp. OH. (**A**) A 10% PAGE gel and zymogram of (**B**) endoglucanase, 1% CMC; (**C**) exoglucanase, 5 µM MUC; (**D**) β-glucosidase, 5 µM MUG; and (**E**) endoxylanase, 1% xylan as substrates. Lane (M), molecular weight markers; lane 1, carboxy-methylcellulose (CMC); lane 2, Avicel (Av); lane 3, oil cake (OC); lane 4, rice husk (Rh); and lane 5, glucose (Gl).

In a complementary approach, the individual components of the secreted enzymes were tested using zymography. The visualization of the 6th day secretome of different cultures (Figure 6A–E) using activity staining shows multiple protein bands with enzyme activities. Endoglucanase and endoxylanase activities were examined by in-gel hydrolysis of CMC and xylan and visualized and de-stained using Congo red solution. The exoglucanase and β-glucosidase fractions were detected using fluorescent substrates MUC and MUG, respectively. The zymogram analysis suggests that the crude fraction from rice husk-grown (Rh, Lane 4) culture had the maximum number of protein bands corresponding to multiple cellulase and xylanase enzymes and high enzyme activities with Rh. Correlating both enzyme activity and zymogram analysis, most of the hydrolytic activities of the strain OH could be associated with these high-intensity bands induced by respective carbon sources.

### 3.7. Production of Secreted Lignocellulolytic Enzymes and Tolerance Profile in the Presence of Chemical Inhibitors

**Figure 7:**
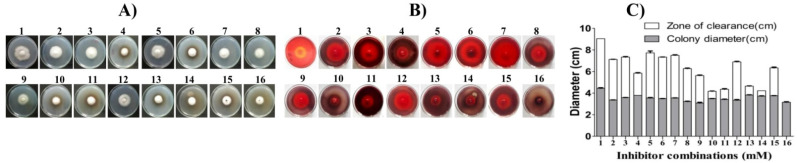
Effect of single and combined inhibitors (IC_50_; mM) on endoglucanase activity of *Colletotrichum* sp. OH. (**A**,**B**) The mycelium growth and endoglucanase activity of OH and (**C**) bar graph showing colony diameter and zone of clearance on 1% CMC agar plate supplemented with different combinations of single and mixed inhibitors (combinations 1–16, Table 1) at their respective IC_50_ concentrations (mM). Experiments were performed in triplicate, and the heights of the bars and the error bars indicate the means ± standard error mean (SEM).

In this study, the impact of the four inhibitors (HMF, LA, HQ, and H_2_O_2_) and their combinations (16, Table 1) on the cellulase expression by the broad-spectrum inhibitor-tolerant and lignocellulolytic *Colletotrichum* sp. OH was further tested. Remarkably, the fungus can grow and simultaneously produce endoglucanase on CMC agar plates amended with an IC_50_ (mM) concentration of different inhibitor combinations, as mentioned in Table 2. As shown in Figure 7A–C, the fungus OH could grow and produce a distinctive clearing zone around its colony on the third day of incubation. The area of clearance on CMC plates amended with single inhibitors HMF, LA, and H_2_O_2_ (combinations 2, 3, and 5; Figure 7) was prominent compared to hydroquinone only (combination 4) and its other combinations (10, 11, 13, and 14), suggesting hydroquinone as an inhibitor for endoglucanase production. Remarkably, in the presence of HMF and H_2_O_2_, the inhibitory effects of HQ decrease in dual combinations 6 and 15. Additionally, triple mixtures with no HQ (combination 9) showed better growth and enzyme production than HQ plates (combinations 10, 11, and 14). The plate-based assay showed the strain OH to be a potent native HMF- and H_2_O_2_-tolerant endoglucanase producer.

**Figure 8:**
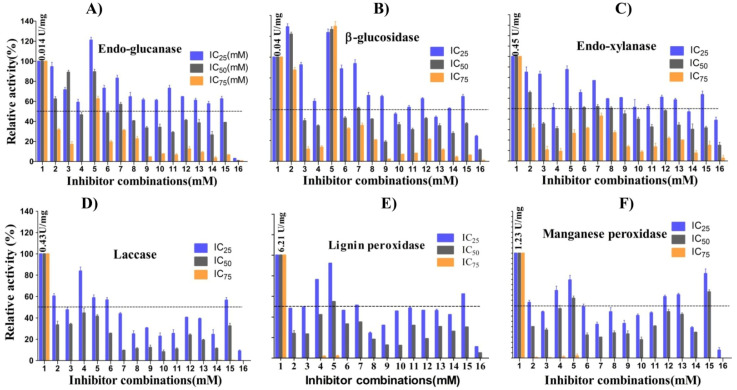
Effect of single and combined inhibitors on the lignocellulolytic activity of *Colletotrichum* sp. OH. (**A**–**F**) The relative activity of OH in the presence of different single and combinations of inhibitors (combinations 1–16) at their respective IC_25_, IC_50_, and IC_75_ (mM) concentrations. The dotted lines show 50% inhibition of different enzymes tested in the presence of inhibitors. The values are in comparison with that of the untreated control. Specific enzyme activities as units per milligram are integrated within combination 1. Experiments were performed in triplicate, and the heights of the bars and the error bars indicate the means ± standard error mean (SEM).

The study’s next step was to characterize the secretome (bulk crude extract) from the strain OH. To do this, we tested the concentrated crude extract from the rice husk-grown cultures for the activities of six enzymes, endoglucanase, BGL, endoxylanase, Lac, Lip, and MnP. The enzyme assays consisted of all sixteen inhibitor combinations (Table 1) and three separate ones, including IC_25_, IC_50_, and IC_75_ (25 mM intervals) concentrations for each inhibitor tested. HMF (8.8, 17.76, and 23.45 mM), LA (13.75, 29.77, and 37.78 mM), HQ (6.5, 10.76, and 17.75 mM), and H_2_O_2_ (25, 50, and 75 mM) were used to test the enzyme activities. Thus, for each enzyme, the activities were tested in 16 combinations with three IC values (48 reactions) and relative activities were compared for tolerance (Figure 8A–F). We selected a stringent cutoff to define the enzyme tolerance threshold as 50% of the no inhibitor activity. Remarkably, the most significant results were the enhanced activities of endoglucanase and β-glucosidase in a single inhibitor reaction mixture, where HMF (combination 2) and H_2_O_2_ (combination 5) were present (Figure 8A,B). The β-glucosidase showed H_2_O_2_-dependent enhancement of activities at all three IC values. The BGL activities increased to 134.2 (75 mM, IC_75_), 129.8 (50 mM, IC_50_), and 125.2 (25 mM, IC_25_) percent compared to no H_2_O_2_. In contrast, the endoglucanase activities were 63.6, 83.9, and 124.0 percent, implying enhancement in only low concentrations of H_2_O_2_ but tolerance toward high H_2_O_2_ levels. In the presence of HMF, the BGL activities were 94 (23.43 mM, IC_75_), 124.3 (17.76 mM, IC_50_), and 132.3 (8.8 mM, IC_25_), implying tolerance and increase in the medium and low HMF concentrations tested. In comparison, the endoglucanase tested showed only tolerance at IC_50_ (60.4%) and IC_25_ (91.5%). Overall, no other enzymes showed activity enhancement in the presence of HMF or H_2_O_2_. Still, tolerance at IC_25_ and IC_50_ levels could be detected for xylanases and ligninase (no tolerance for HMF at IC_50_) (Figure 8C–F). In the dual combination 7 (HMF + H_2_O_2_) for IC_50_ and IC_25_, the activities decreased to 58.8 and 82.5% for endoglucanase, 50.0 and 95.0% for BGL, and 53.7 and 75.6 for xylanases, suggesting a moderate tolerance for mixed inhibitors at a low concentration. The activity reduction of the above three enzymes with HQ alone (combination 4, IC_25_) was negated by dual combinations 6 (HMF + HQ) and 15 (HQ + H_2_O_2_) (Figure S8). Remarkably, the endoglucanase activity was 75% in the triple combination 11, IC_25_ (HMF + HQ+ H_2_O_2_), compared to individual HQ activity of 58.7%. The fourth chemical, levulinic acid (combination 3), was the most tolerated chemical by OH, for which the endoglucanase activity was higher at 29.77 mM (IC_50_; 82.5%) than 13.75 mM (IC_25_; 64%).

The BGL and xylanase activities in the presence of 13.75 mM LA were 91 and 82.6%, respectively, suggesting that the enzymes tolerate IC_25_ values. Several triple IC_25_ combinations (9, 11, and 14) showed activities higher than 50% compared to no inhibitor, but only xylanases in triple combination 9 (IC_50_; HMF + LA + H_2_O_2_) showed activity reaching 59%. For the three ligninases (laccase, lignin peroxidase, and manganese peroxidase), there was tolerance for various combinations but no enhancement of activities was detected (Figure 8D–F; Figure S9). Additionally, unlike cellulases, ligninase activity was not detected in IC_75_ combinations. Laccase activities tolerated IC_25_ individual HQ (84.5%), HMF, and H_2_O_2_ (61.6%), and dual combinations HQ + H_2_O_2_ (67.1%), and HMF + HQ (57.1%). LiP activities tolerated individual combinations H_2_O_2_ (92.2 and 54.7% in 25 and 50 mM), HQ (76.2%, 6.5 mM), HMF (53%, 8.8 mM), and LA (53%, 13.75 mM), several double combinations, and one triple combination (Figure S9). Manganese peroxidase was most tolerant to a dual combination of HQ+ H_2_O_2_ (80.4 and 65.2% in IC_25_ and IC_50_, combination 15) followed by H_2_O_2_ (75.1 and 57.4% in 25 and 50 mM), HQ (65% in 6.5 mM), and HMF (54.5% in 8.8 mM). Interestingly, the inhibitory effect of individual LA (44.3% in 13.75 mM) was reduced in dual combinations LA + HQ (62.3%) and LA+ H_2_O_2_ (60.7%). Overall, the data suggest that quadruple combination 16 at all IC values led to a significant decrease in cellulase and xylanase activities with complete inhibition of ligninases.

### 3.8. Stability of β-Glucosidase in the Presence of Exogenous H_2_O_2_

**Figure 9:**
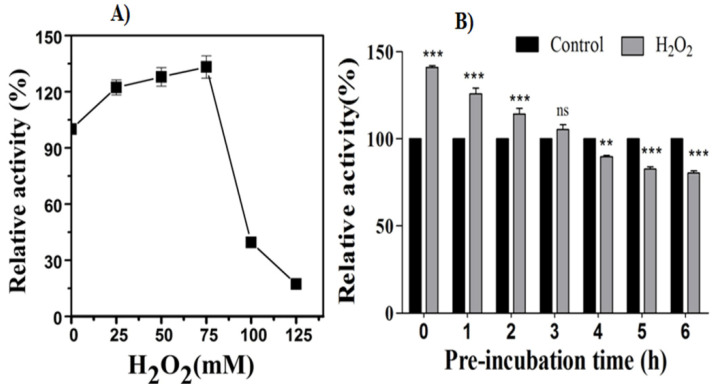
Effect of H_2_O_2_ on β-Glucosidase activity. (**A**) Effect of different concentrations of H_2_O_2_ (25, 50, 75, 100, 125 mM) on β-Glucosidase activity. The black boxes represent relative BGL activity with respect to no H_2_O_2_. The error bars indicate the means ± standard error mean (SEM) from three independent experiments, and (**B**) pre incubation has advantages in enhancing BGL activity in the presence of exogenous H_2_O_2_. H_2_O_2_ tolerance of BGL activity was investigated by adding H_2_O_2_ to bulk crude enzyme at different time points (0–6 h). The 0 mM H_2_O_2_ pre incubation condition in this experiment was crude enzyme incubated in buffer without any H_2_O_2_. BGL activity without H_2_O_2_ during 0–6 h pre incubation was defined as control (100%). The significant difference (**) was set at *p* < 0.01, and the highly significant difference (***) was set at *p* < 0.001 in Tukey’s *t*-test, compared to the 0% H_2_O_2_ pre incubation control. Values are the mean ± SEM of results from three independent experiments.

Among the tested enzymes, β-glucosidase showed activity enhancement in the presence of H_2_O_2_. Next, the β-glucosidase activity and stability were tested using higher H_2_O_2_ concentrations. β-glucosidase activity further increased in 75 mM H_2_O_2_, while at 100 and 125 mM, activities declined significantly (Figure 9A). Further, BGL stability was tested by incubating the crude extract with buffered 75 mM H_2_O_2_ for 0–6 h before adding substrate. Remarkably, the addition of 75 mM H_2_O_2_ enhanced the activity by 1.44-fold compared to no H_2_O_2_ in the 0 h reaction (Figure 9B). The bulk extract BGL remained stable up to a 3 h pre incubation, when the activity reached ~1.08-fold that of control. The BGL activity after a 3 h pre incubation showed a decrease at a uniform rate (~0.9-fold/hour), reaching 82% of the no H_2_O_2_ condition in a 6 h incubation. The 6 h H_2_O_2_ incubated bulk extract showed activity reduction by 57.7% compared to 0 h H_2_O_2_, suggesting enzyme deactivation.

## 4. Discussion

The major impediment in realizing lignocellulosic biomass as a low-cost resource to produce biofuel is the resistant properties, which require costly pretreatment strategies. Different biomass pretreatments aid in making several biopolymers accessible for the lignocellulolytic enzymes. However, pretreatments result in a multitude of chemical compounds, which inhibit enzymatic saccharification and microbial growth and fermentation [7,10]. However, efficient lignocellulosic biomass enzymatic depolymerization or conversion to valuable chemical products relies on biomass pretreatments [8]. Thus, lignocellulose conversion to monomeric sugars or beneficial chemical compounds depends on biocatalysts harboring tolerance toward biomass pretreatment chemicals and their by-products.

Endophytic fungi are a rich source of enzymes for the degradation of cellulose, hemicelluloses, pectin, and lignin. Since the endophytes are constantly evolving inside the host tissue, the biomolecules they produce mimic host physiology and adaptations [76].

In this study, we screened *Ocimum sanctum* leaf fungal endophytes for their lignocellulolytic enzyme production. The preliminary screening showed that one native endophytic fungal strain coded OH appeared to be a promising lignocellulolytic producer. The potential lignocellulolytic-producing fungus identified as *Colletotrichum* sp. OH is a member of the family Glomerellaceae in Ascomycota [78]. The genus *Colletotrichum* groups most species into 14 complexes, and many species complexes are known to have endophytic lifestyles [79]. Chowdhary and Kaushik [45] have also isolated endophytic *Colletotrichum* sp. (KR017032) from *O. sanctum*. Several endophytic strains of the genus *Colletotrichum* have biotechnological applications such as biodiesel production, mycoherbicide, plant growth promotion, agarwood formation, and as a source of novel biocatalyst [74,76,77].

The exciting feature of the potential lignocellulolytic fungus *Colletotrichum* sp. OH was its tolerance to lignocellulose pretreatment and pretreatment-derived compounds. Different lignocellulose pretreatment methods generate various inhibitory compounds that could affect subsequent fermentation (inhibiting the microorganism’s growth and survival) and enzymatic hydrolysis (inhibiting enzymes). The generation of these compounds depends on the lignocellulosic source, the type of pretreatment used, and the conditions employed in pretreatment [80,81,82]. Here, in this study, *Colletotrichum* sp. OH was tested for tolerance on solid media and liquid media with different inhibitory compounds. The results showed that *Colletotrichum* sp. OH has a spectrum of tolerance and limits to inhibitory compounds. Among the tested aldehydes (vanillin, *p*-hydroxybenzaldehyde, 5-hydroxymethylfurfual; HMF, and furfural), HMF was most tolerated while vanillin and *p*-hydroxybenzaldehyde were the least tolerated. The growth of strain OH was reduced to approximately 50% at 5 mM of vanillin and *p*-hydroxybenzaldehyde, while complete growth inhibition occurs at higher values, which was same for the IC_50_ reported in oleaginous yeast *Trichosporon fermentans* [83]. Klinke et al. and Chen et al. [84,85] reported that vanillin and PHB were toxic to fungi at 0.1 mM and occur in corn stover hydrolysate at a concentration of 0.060 and 0.076 mM, respectively. The relative biomass growth of OH was ~59% on 10 mM furfural, and complete growth inhibition occurred in 15 mM conditions. Ruan et al. [86] reported the normal growth of *Mortierella isabellina* on 2.5 g/L HMF and 1 g/L furfural, which is in line with the reported OH tolerance.

Interestingly, the fungus showed maximum tolerance to HMF, where growth reduction was only above 15 mM and growth stopped beyond 20 mM. HMF typically interrupts electron transfer and hence operates on glycolysis and the TCA cycle, affecting the energy metabolism of microorganisms [87]. HMF in ammonia fiber expansion-pretreated corn stover and dilute acid switchgrass hydrolysate is 0.6 and 1.7 mM, respectively [88]. Interestingly, in our study, HMF had little impact on OH growth at this concentration. Remarkably, the IC_50_ of HMF (17.5 mM) was significantly higher than the value reported for *Pleurotus ostreatus* (12.5 mM; IC_50_) by Feldman et al. [89]. It is reported that several endophytic fungi can tolerate and utilize HMF better than furfural in a 5 mM medium [90]. In addition to aldehydic and acidic inhibitors, the phenolic compounds primarily produced from lignin degradation are present in biomass hydrolysates at varying concentrations [85]. Different acidic inhibitors belonging to furan or aromatic organic acids (*p*-hydroxybenzoic, ferulic, syringic, vanillic, furoic, and gallic acid) and aliphatic acids (acetic, formic, levulinic, and caproic acid) were abundant in the lignocellulosic hydrolysate [11,12,80].

In the second group of pretreatment-derived chemicals tested, the most tolerated chemicals were levulinic acid (29.77 mM, IC_50_), gallic acid (25.87 mM, IC_50_), and formic acid (15.87 mM, IC_50_). In contrast, the least tolerated, *p*-hydroxybenzoic acid, was toxic at low concentrations (5 mM), where the relative growth was reduced by more than 60%. Similarly, *Fusarium oxysporum* f. sp. *niveum* mycelial mass was reduced by 63.7% at 1600 mg L^−1^ (11.6 mM) of *p*-hydroxybenzoic acid [91]. With levulinic acid, a weak acid, and an HMF derivative, the relative growth of the strain OH at a 20 mM concentration approached 50% on day 7, roughly ten times that reported for *Trametes versicolor* [92]. Levulinic acid was slightly more inhibitory than acetic acid and affects the uptake of xylose and mannose [92]. In corn stover and spruce hydrolysate, the concentrations of levulinic and formic acid were only around 0.3 mM (0.13–0.4 g/L) and 0.17 mM (0.2–0.8 g/L), respectively [93,94]. At these concentrations, the growth inhibition of *Colletotrichum* sp. OH was 4% and 2%, respectively. The inhibitory effects of weak acid including LA is well studied in highly fermentative yeast. LA interferes with mitochondrial function by inhibiting the cytochrome biosynthesis pathway in *Saccharomyces cerevisiae* and *Candida utilis* by inhibiting the enzyme *δ*-aminolevulinic acid dehydratase [95]. Additionally, by binding to protons in the cellular environment, LA impairs the translocation of protons across the membranes, in turn increasing ATP demand and redox oxidative stress [92,96]. The highly fermentative *Saccharomyces cerevisiae* was able to tolerate 400 mM LA in the aerobic glucose growth phase with an increased lag phase [96]. The high tolerance of *Colletotrichum* sp. OH (29.77 mM, IC_50_ or 3.5 g/L) as compared to *Trametes versicolor* (0.3 g/L) may be due to its high redox tolerance.

The gallic acid tolerance concentration for *Colletotrichum* sp. OH reported here is ~10 times more than the tested concentration for *Fusarium oxysporum* *f.* sp. *Niveum* [97]. However, the reported IC_50_ values for the growth of oleaginous yeast *Trichosporon fermentans* on gallic acid was 10–15 times more than that of *Colletotrichum* sp. OH [98].

As in our study, several phenolics, *trans*-cinnamic acid, ferulic acid, and *p*-coumaric acid, in the 5–50 mM range inhibited *Colletotrichum* sp. with the least tolerated phenolic being *trans*-cinnamic acid [99]. In 10 mM *p*-coumaric acid, the tolerance of *Colletotrichum* sp. OH was slightly better than hydroquinone. However, the strain OH can tolerate hydroquinone until 15 mM (relative growth 50%). Adeboye et al. [100] reported that 10 mM of *p*-coumaric and hydroquinone was too toxic for the growth of *Saccharomyces cerevisiae.*

The other groups of chemicals tested included hydrogen peroxide and ionic liquids, which are pretreatment chemicals. The use of oxidants for pretreatment in ozonolysis, wet oxidation, and alkaline peroxide pretreatment (AHP) reduces the cellulose crystallinity and disrupts linkages of carbohydrates and lignin [12]. The use of hydrogen peroxide at pH 11.5 (AHP) is an effective pretreatment of lignocellulosic biomass in the context of ethanol production [101]. The screening of strain OH depicted a remarkable tolerance efficacy for H_2_O_2_ compared to other fungi reported in the literature, viz., *Colletotrichum gloeosporioides* [102] and *Colletotrichum higginsianum* [103], and this fungus represents a potential candidate for testing in the processes of saccharification followed by AHP.

Moreover, the use of ionic liquids (ILs) is a green method for pretreatment of lignocellulose materials [104]; however, residual ILs in pretreated slurries have remarkable toxicity for fermentative microorganisms and enzymes [105]. ILs are toxic to biomacromolecules and completely blocked the growth of well-known fungus *F. oxysporum* BN with 5% (Cl^−^)-based ILs [106]. The fungus OH shows high tolerance (50% growth inhibition at the above mentioned concentration of the IL). The data of the cell wall stress toleration phenotype of the reported fungus demonstrate its inherent extremophile phenotype. The strain OH could tolerate and degrade Congo red dye at a 1 mg/mL concentration. To the best of our knowledge, this is the highest CR concentration tolerated by any fungus. Recently, a survey of diverse fungi for CR-associated cell wall stress tolerance identified *Aspergillus niger* and *Trichoderma atroviride* as candidates tolerating 600 µg/mL CR [107].

Next, in this study, to account for the complex synergistic effect of various inhibitors present in the lignocellulosic hydrolysates [108], we tested 16 combinations of four chosen chemicals. Ruan et al. reported a more substantial inhibition on biomass, lipid production, and carbon source utilization when four inhibitors (furfural, HMF, ferulic acid, and coumaric acid) were mixed and added to the culture of *Mortierella isabellina* compared to cultures with individual inhibitors [86]. A few promising fungi tolerant to either single or mixed inhibitors are well characterized (reviewed in [11,16]) [109]. Evidently, no literature is available regarding the synergistic effect of pretreatment-derived inhibitors with H_2_O_2_ on fungal growth and metabolism. However, using statistical methods, a mixed concentration of 3.3 mmol/L formic acid, 21.6 mmol/L levulinic acid, and 6.6 mmol/L furfural inhibitors was optimized to increase the growth and ethanol yield of *Saccharomyces cerevisiae* [110]. Similarly, the fungus *T*. *versicolor* was reported to grow on mixed inhibitors of phenol, levulinic acid, HMF, and furfural at 0.4, 0.3, 0.2, and 0.6 g/L, respectively, by detoxification of the inhibitors [92]. The degradation of the inhibitors was associated with the production of the enzymes laccase, peroxidase, manganese peroxidase, and phenol oxidase. The growth inhibition of the strain OH was 51.67% (HMF alone), 50% (LA alone), and only reduced to 50% in the LA and HMF mixed condition (combination 8, Table 3) Since *Colletotrichum* sp. OH was able to produce inhibitor-tolerant laccase and manganese peroxidase (Figure 5F and Figure 8), an identical mechanism of LA and HMF degradation or their modification into less toxic derivatives might be operational in the fungus. The reduced relative growth and biomass accumulation of OH on the supplemented media (a combination of HMF, levulinic acid, hydroquinone, and hydrogen peroxide) could be due to the additive effect of the multiple inhibitors. Further, the dual mixtures showed a minor inhibition increase as single combinations, pointing to an adaptive response. Remarkably, the dual combinations where H_2_O_2_ was a partner showed marginally better or lower biomass inhibition than H_2_O_2_ alone, suggesting an adaptive cross-tolerance response. Thus, the reported fungus is better adapted to tolerate single and double combinations than triple combinations where more complex synergistic effects come into play.

The tolerance of single or multiple furan, acidic, and phenolic inhibitors by this fungus may be due to its capacity to degrade or metabolize the inhibitors, as reported for several filamentous fungi and yeast (reviewed in [26]) [92]. The exact mechanism of tolerance to all inhibitors by the members of the *Colletotrichum* genus is unknown; possibly, they can degrade phenolic inhibitors [111] or are inherently tolerant [112]. However, further experimental data with the fungus OH are required to understand the stress tolerances response.

Similarly, the tolerance toward extracellular H_2_O_2_ may be due to the presence of robust antioxidant enzymes such as catalase, glutathione reductase, superoxide dismutase, and high glutathione content to reduce oxidative stress damage [113]. The transcriptome of the fungus *Colletotrichum lupini* showed upregulation of catalase and peroxidase genes in response to plant infection [114]. The secretome analysis of *Colletotrichum graminicola, Colletotrichum tabacum,* and *Colletotrichum destructivum* reported the expression of H_2_O_2_-producing copper radical–alcohol oxidase [115], suggesting the presence of peroxidases in the genome of *Colletotrichum* species.

In endophytes and other fungi, their capacity to live in a host environment depends on the composition and function of its cell wall as it activates numerous pathways in response to stress stimuli, such as oxidative, pH, or cell wall damage. One such pathway, the cell wall integrity (CWI) signal transduction pathway, regulates gene expression of cell wall biosynthesis and the production of secondary metabolites for stress tolerance [116]. The fungus OH cell wall tolerance hints that a hyperactive CWI might be operational, considering that the fungus was isolated from antioxidant-rich *Ocimum* leaves. Therefore, the endophyte produces lignocellulolytic enzymes naturally tolerant to oxidants as an adaptive response.

A few promising fungi tolerant to either single or mixed inhibitors are well characterized (reviewed in [11,16,109]).

As only handful of mixed inhibitor tolerant fungi are reported (reviewed in [11,16]), new and novel fungal isolates from diverse ecosystems for improved bioabatement of inhibitors are desirable [25]. Moreover, most studies test low concentrations of the inhibitors or focus only on detoxification or its associated mechanisms [11,16]. Furthermore, plant biomass-degrading enzymes or their tolerance is not well studied. Thus, we tested the lignocellulolytic potential and tolerance of several secretory enzymes of *Colletotrichum* sp. OH.

Using natural and commercial carbon sources, induction of expression and significant yields for multiple consortia of cellulase, hemicellulase, pectinase, and ligninase enzymes were observed from the fungus *Colletotrichum* sp. OH. These enzymes play a role in fungal metabolism and survival in rapidly shifting environments such as endophytes. The production of the maximal amount of endoglucanase activity in the presence of CMC is known in *T. reesei* RUT-C30 [117]. The enhancement of exoglucanase activities in the presence of rice husk was in line with the report of Liu et al. [118] in utilizing rice straw as a sole carbon source for *Aspergillus fumigatus* Z5. The OH strain maintained a lower level of enzyme production in the presence of glucose as a carbon source, suggesting a weak glucose catabolic repression activity, and similar glucose induction was observed in *Sporothrix schenckii* as well [119]. Moreover, this study shows similar lignocellulolytic production titers to other endophytic strains reported in the literature, i.e., *Acremonium* sp. [120], *Alternaria* sp. and *Colletotrichum* sp. DM06 [121], *Fusarium* sp. QH101 [122], and *Colletotrichum lindemuthianum* [123]. The observed consortium of lignocellulase titers in OH could be due to the strain’s inherent genetic capacity and the use of different medium and substratum compositions, which play a significant role in the production of enzymes [64]. In line with this argument, the secretome of *Colletotrichum lupini* is reported to consist of 28 secreted carbohydrate-active enzymes (CAZymes) (cited above) while the secretome of *C. graminicola, C. tabacum,* and *C. destructivum* consisted of 29–52% CAZymes out of 400 detected proteins (cited above). In parallel, the extracellular lignocellulolytic enzyme secretome was analyzed using zymography. The multiple protein and enzyme bands in PAGE and zymography point to a few lignocellulolytic and xylanase enzyme systems present as families, subfamilies, or isoform levels.

The essential objective in advancing second-generation biofuels is to develop a robust microbial host that is well endowed with significant levels of lignocellulolytic enzymes and the capacity to overcome or detoxify broad-spectrum chemical inhibitors. Additionally, new saccharification methods using innovative biocatalysts that possess better characteristics for commercial use are needed. Therefore, natural or engineered enzymes that have high biocatalyst efficiency and tolerance to lignocellulosic generated by-products or residual pretreatment chemicals are currently needed. In this line, the study tested the strain OH’s ability for simultaneous growth and endoglucanase production on solid CMC amended with inhibitor combinations at IC_50_ values. The semi-quantitative method suggests that hydroquinone alone acts as an inhibitor for endoglucanase production. This inhibitory effect of HQ alone is reduced with its dual combination with HMF and H_2_O_2_.

Additionally, in triple mixtures with no HQ (combination 9), better growth and enzyme production than HQ plates (combinations 10, 11, and 14) suggest HQ-mediated gene expression repression. Noticeably, exogenous hydroquinone but not H_2_O_2_ represses the expression of manganese peroxidase gene expression in *Ceriporiopsis subvermispora* [124]. Nevertheless, the effects of hydroquinone, HMF, H_2_O_2_, and their combinations on the expression of cellulases and hemicellulase are not well proven. Strain OH gene expression analysis may shed light on several observed phenotypes and elucidate pathways for the simultaneous development of tolerance and enzyme secretion. The work presented in this section suggests that the fungus was a multi-inhibitor-tolerant microorganism with enzyme production ability in the presence of inhibitors.

We further tested the tolerance of the secretome produced from the rice husk culture to 16 inhibitor combinations in three different concentrations. The data on the tolerance of the enzyme to the inhibitor combinations and the strain’s growth profiles were similar, indicating that the endophyte fungus *Colletotrichum* sp. OH is an extremophile capable of secreting inhibitor-tolerant proteins. Among the enzymes studied, only endoglucanase and β-glucosidase demonstrated enhancement of activities and tolerance toward the tested inhibitors. Although the enhancement and tolerance were inhibitor and concentration specific, broadly, the endoglucanase, β-glucosidase, and xylanase secreted by this fungus can tolerate higher inhibitor concentrations than the three ligninases (Figure S9**)**. The key findings include increasing β-glucosidase and endoglucanase activity in the presence of HMF and H_2_O_2_, enzyme activities tolerant to inhibitors and their combinations, and reduction in hydroquinone and levulinic acid inhibitory effects on enzymatic activities by HMF and H_2_O_2_. Laccase, MnP, and LiP tolerated H_2_O_2_, HQ, HMF, and their combinations at low concentrations.

β-glucosidase revealed a redox tolerance profile as when the reaction consisted of 25, 50, and 75 mM H_2_O_2_, the H_2_O_2_-dependent activities increased by 1.25-, 1.29-, and 1.34-fold, respectively, compared to a no H_2_O_2_ control. The brown-rot fungus *Postia placenta* MAD 698-R was documented to secrete an oxidation-tolerant bulk enzyme fraction as an adaptation to the wood degradation process that employs reactive oxygen species (ROS) [125]. As in our report on the effects of peroxide in the enhancement of β-glucosidase activity, the enzymes secreted by *Postia placenta* and *Trichoderma reesei* RUT-C30 have similar H_2_O_2_ tolerance and hydrolysis enhancement activities. Similarly, an increase in endoglucanase (CMCase) in 25 mM H_2_O_2_ and a decrease in xylanase activities in the presence of H_2_O_2_ were observed. The activity data with other inhibitors underscored the remarkable tolerance limits of β-glucosidase produced by this fungus. Remarkably, the activity of β-glucosidase increased by 1.32- and 1.24-fold in the presence of HMF (8.8 and 17.6 mM), marginally decreased by 0.94-fold in 23.45 mM HMF, was reduced by 0.95-fold in HMF + H_2_O_2_ (8.8, 25 mM), and decreased to 0.91-fold in 13.75 mM LA. The marginal β-glucosidase activity could be detected in four inhibitor combination reactions, suggesting a wide range of tolerance. Alves et al. [126] reported a soil metagenome-derived β-glucosidase (Laf2) with multiple inhibitor tolerance and enhancement of its activity by 70% in the presence of 1% HMF (7.93 mM). However, ours is the first report with HMF and H_2_O_2_ stimulation of both endoglucanase and β-glucosidase activities.

The benzene-derived phenolic compound, hydroquinone (1,4-Benzenediol), is reported at a low concentration range in different acid hydrolysates, e.g., in Norway spruce (17 mg/L) [127] and was considered less toxic to *S. cerevisiae* (~10 mM) than its laccase-oxidized derivative benzoquinones [127]. Among the white-rot basidiomycete fungi, HQ causes concentration-dependent changes in cellular morphology in *Ceriporiopsis subvermispora* (maximum 0.75 mM) [124], while complete growth inhibition occurs in *Cerrena unicolor* IBB 300 and *Trametes versicolor* IBB 775 in 0.5 mM of hydroquinone [128]. On the other hand, brown-rot fungi produce methoxylated or methylated hydroquinone derivatives to drive Fenton reaction-mediated lignin depolymerization [129]. They also act as redox intermediates in activating lytic polysaccharide monooxygenase (LPMO) enzymes for oxidative cellulose degradation in different fungi [129,130]. The tolerance of hydroquinone and other benzene derivative phenolics *in S. cerevisiae* involves cytoskeletal and oxidative stress tolerance, correct regulation of iron homeostasis, and vacuolar ATPase stress [131]. A similar orthologous tolerance network might exist in the strain OH for hydroquinone tolerance, which needs further experimental validation. HQ supplementation in some fungi can act as an activator or repressor of lignocellulolytic enzymes depending on the fungi and enzymes [124]. Our enzyme assay data suggest that the activities of cellulases and xylanase reduced to 50% in the presence of 6.5 mM hydroquinone, while the ligninolytic enzymes show a high tolerance in the same concentration. At a 5 mM concentration, Avicel hydrolysis by Novozymes cellulase mix was unaffected [132].

Further, the inhibitory effect of HQ in the enzymatic reactions of cellulases and xylanase activity decreased in the presence of HMF (combination 4 vs. 6, Figure 8, Figure S8). The 5-hydroxymethylfurfural-mediated activity enhancement discussed above could be due to HMF’s protein-stabilizing function in line with its known covalent hemoglobin (Hb) modification. Structural data suggest that binding of HMF to Hb’s N-terminal alpha valine nitrogen moves the allosteric equilibrium to the Hb oxygen affinity state as well [133]. However, similar speculative binding of HMF and stabilizing of the lignocellulolytic enzymes could be an explanation for enhancement of various activities in the presence of HMF.

β-glucosidase is a critical enzyme for complete hydrolysis of cellulosic biomass as it produces glucose from oligosaccharides and cellobiose. The stability of this enzyme toward operational conditions is a critical parameter in industrial settings [134,135]. The 3 h incubation of the bulk crude enzyme with 75 mM of H_2_O_2_ showed 1.34-fold enhancements in BGL activity, suggesting a high tolerance with potential application in oxidative pretreatment conditions. An efficient cellulase enzyme cocktail from the secretome of the strain OH can be envisioned based on the simultaneous activity enhancement of endoglucanase along with β-glucosidases.

The data presented in this work suggest several future experiments to elucidate the inhibitory tolerance mechanisms operational in this fungus using genomics and proteomics approaches. These studies will help better understand the intracellular pathways and extracellular players operational in cell wall perturbation and oxidative and chemical inhibitor tolerance phenotypes of *Colletotrichum* sp. OH. The increased production and compositional analysis of the lignocellulolytic enzyme titers using medium and culture condition optimization will help in using the secretome in plant biomass degradation studies relevant to biorefinery industries. An in-depth understanding of the role of pretreatment-derived chemical inhibitors in CAZyme expression regulation by the fungus is needed to support our data for hydroquinone-mediated repression of endoglucanase production and de-repression by H_2_O_2_ and HMF. Further, the industry-friendly crude enzyme secretome or purified β-glucosidase and endoglucanase need to be addressed in terms of the inherent mechanisms of inhibitor tolerance and activity enhancements based on individual protein sequences and structure–function analysis.

## 5. Conclusions

In this study, we found that the *Ocimum sanctum* L. leaf endophytic fungus *Colletotrichum* sp. OH can tolerate and grow on various chemical stressors. The fungus grew and tolerated single and mixed chemicals from furan, acidic, and phenolic groups known to exist in the plant biomass pretreatment process. The fungus exhibited very high tolerance to hydrogen peroxide and cell wall-perturbating agent Congo red. The native *Colletotrichum* sp. OH can secrete enzymes for lignin, cellulose, and hemicellulose depolymerization. Notably, the secreted lignocellulolytic enzyme activities were either tolerant or enhanced in the presence of chemical inhibitors and H_2_O_2_. The enhancement of β-glucosidase activities in the presence of H_2_O_2_ (25, 50, and 75 mM) and HMF (8.8 and 17.76 mM) and tolerance to their combinations suggests an inherent trait of oxidative tolerance. Thus, the study highlights the potentials of *Colletotrichum* sp. OH to address bottlenecks of pretreatment inhibitors and can serve as a robust platform for lignocellulosic-based bioprocesses in future studies.

## Figures and Tables

**Table 1:** Combinations of four different inhibitors at their IC_50_ concentration.

Inhibitors IC_50_ ^a^ (mM)				
Combination	HMF	LA	HQ	H_2_O_2_
1	−	−	−	−
2	+	−	−	−
3	−	+	−	−
4	−	−	+	−
5	−	−	−	+
6	+	−	+	−
7	+	−	−	+
8	+	+	−	−
9	+	+	−	+
10	+	+	+	−
11	+	−	+	+
12	−	+	−	+
13	−	+	+	−
14	−	+	+	+
15	−	−	+	+
16	+	+	+	+

^a^ Molar concentration of 50% inhibition on the growth of OH. + Presence, − Absence.

**Table 2:** The concentration of inhibitors required to inhibit the growth of OH to 50%.

Inhibitors	IC_50_ ^a^ (mM)
**Aldehyde**	
5-HMF	17.76
Furfural	8.39
Vanillin	5.03
*p*-Hydroxybenzaldehyde	5.11
**Acid**	
Levulinic acid	29.77
Gallic acid	25.87
Formic acid	15.87
*p*-Hydroxybenzoic acid	4.94
**Phenolic**	
Hydroquinone	10.76
*P*-Coumaric acid	8.63
**Oxidative stress**	
Menadione	0.123
Hydrogen peroxide	50

^a^ Molar concentration of 50% inhibition on the growth of OH.

**Table 3:** Effect of inhibitor combinations on mycelial growth and biomass accumulation of *Colletotrichum* sp. OH.

Samples	RGI (%) ^a^	Biomass (g/L)	Inhibitor IC_50_
Combination 1	100 ± 0	9.12 ± 0.04	None
Combination 2	51.67 ± 0.5 ^c^	4.66 ± 0.0 ^d^	HMF
Combination 3	50 ± 0.5 ^c^	6 ± 0.02 ^c^	LA
Combination 4	49.17 ± 0.07 ^d^	5.3 ± 0.03 ^c^	HQ
Combination 5	50.56 ± 0.3 ^c^	4.16 ± 0.05 ^d^	H_2_O_2_
Combination 6	46.12 ± 0.05 ^d^	3.6 ± 0.01 ^d^	HMF + HQ
Combination 7	47.22 ± 0.25 ^d^	4 ± 0.01 ^d^	HMF + H_2_O_2_
Combination 8	50 ± 0.05 ^c^	4.16 ± 0.35 ^d^	HMF + LA
Combination 9	38 ± 0.05 ^d^	2.6 ± 0.01 ^e^	HMF + LA + H_2_O_2_
Combination 10	33.33 ± 0.02 ^d^	2.8 ± 0.046 ^d^	HMF + LA + HQ
Combination 11	35 ± 0.005 ^d^	2.06 ± 0.05 ^e^	HMF + HQ + H_2_O_2_
Combination 12	44 ± 0.15 ^d^	4.3 ± 0.02 ^d^	LA + H_2_O_2_
Combination 13	45 ± 0.05 ^d^	3.9 ± 0.02 ^d^	LA + HQ
Combination 14	40 ± 0.15 ^d^	2.6 ± 0.02 ^e^	LA + HQ + H_2_O_2_
Combination 15	46 ± 0.15 ^d^	4.2 ± 0.01 ^d^	HQ + H_2_O_2_
Combination 16	23 ± 0.15 ^e^	N/D ^b^	HMF + LA + HQ + H_2_O_2_

Relative mycelial growth inhibition (RGI) and biomass of *Colletotrichum* sp. OH in the presence of inhibitor combinations. ^a^ RGI (%) = 100%—relative growth in the presence of inhibitors. ^b^ N/D, no growth. IC_50_, Inhibitory Concentration defined as molar concentration for 50% growth inhibition. Values are expressed as the means (*n* = 3) ± SEM. Statistical differences were analyzed by one-way analysis of variance (ANOVA). Significant differences (*p* < 0.05, followed by Tukey’s test) compared with the control are indicated by different letters (^c–e^).

## Data Availability

All data related to this manuscript are incorporated in the manuscript only.

## Supplementary Materials

The following are available online at https://www.mdpi.com/article/10.3390/jof7100785/s1, Figure S1: Uncompressed rooted maximum likelihood phylogenetic tree using *ITS* gene sequences, Figure S2: Rooted neighbor-joining phylogenetic tree based on sequences of *LSU* gene, Figure S3: Growth of *Colletotrichum* sp. OH on different carbon sources, Figure S4: The growth patterns of *Colletotrichum sp*. OH at different pH levels (4–13) at 30 °C, Figure S5: Cell wall stress-tolerant capability of *Colletotrichum* sp. OH, Figure S6: Growth profile of *Colletotrichum* sp. OH on commercial media and agro-waste at 30 °C, Figure S7: Pectinase activity of *Colletotrichum* sp. OH in the presence of 1% citrus pectin, Figure S8: Relative enzyme activity of endoglucanases (EnG), β-glucosidases (BGL), and xylanases (XLN) in the presence of lignocellulose-derived inhibitor combinations, Figure S9: Relative enzyme activity of laccases (Lac), lignin peroxidases (LiP), and manganese peroxidases (MnP) in the presence of lignocellulose-derived inhibitor combinations.

## References

[1] Agbor V., Cicek N., Sparling R., Berlin A., Levin D. (2011). Biomass Pretreatment: Fundamentals Toward Application. Biotechnol. Adv..

[2] Clauser N., González G., Mendieta C., Kruyeniski J., Area M., Vallejos M. (2021). Biomass Waste as Sustainable Raw Material for Energy and Fuels. Sustainability.

[3] Cai J., He Y., Yu X., Banks S., Yang Y., Zhang X., Yu Y., Liu R., Bridgwater A. (2017). Review of Physicochemical Properties and Analytical Characterization of Lignocellulosic Biomass. Renew. Sustain. Energy Rev..

[4] Himmel M.E., Ding S.Y., Johnson D.K., Adney W.S., Nimlos M.R., Brady J.W., Foust T.D. (2007). Biomass recalcitrance: Engineering plants and enzymes for biofuels production. Science.

[5] Gupta V.K., Kubicek C.P., Berrin J.G., Wilson D.W., Couturier M., Berlin A., Filho E.X.F., Ezeji T. (2016). Fungal Enzymes for Bio-Products from Sustainable and Waste Biomass. Trends Biochem. Sci..

[6] De Bhowmick G., Sarmah A.K., Sen R. (2018). Lignocellulosic biorefinery as a model for sustainable development of biofuels and value added products. Bioresour. Technol..

[7] Hendriks A.T., Zeeman G. (2009). Pretreatments to enhance the digestibility of lignocellulosic biomass. Bioresour. Technol..

[8] Mosier N., Wyman C., Dale B., Elander R., Lee Y.Y., Holtzapple M., Ladisch M. (2005). Features of promising technologies for pretreatment of lignocellulosic biomass. Bioresour. Technol..

[9] Yoo C.G., Meng X., Pu Y., Ragauskas A. (2020). The critical role of lignin in lignocellulosic biomass conversion and recent pretreatment strategies: A comprehensive review. Bioresour. Technol..

[10] Palmqvist E., Hahn-Hägerdal B. (2000). Fermentation of lignocellulosic hydrolysates. II: Inhibitors and mechanisms of inhibition. Bioresour. Technol..

[11] Kim D. (2018). Physico-Chemical Conversion of Lignocellulose: Inhibitor Effects and Detoxification Strategies: A Mini Review. Molecules.

[12] Jonsson L.J., Martin C. (2016). Pretreatment of lignocellulose: Formation of inhibitory by-products and strategies for minimizing their effects. Bioresour. Technol..

[13] Kumar V., Yadav S.K., Kumar J., Ahluwalia V. (2020). A critical review on current strategies and trends employed for removal of inhibitors and toxic materials generated during biomass pretreatment. Bioresour. Technol..

[14] Palmqvist E., Hahn-Hägerdal B. (2000). Fermentation of lignocellulosic hydrolysates. I: Inhibition and detoxification. Bioresour. Technol..

[15] Ko J.K., Um Y., Park Y.-C., Seo J.-H., Kim K.H. (2015). Compounds inhibiting the bioconversion of hydrothermally pretreated lignocellulose. Appl. Microbiol. Biotechnol..

[16] Sodré V., Vilela N., Tramontina R., Squina F.M. (2021). Microorganisms as bioabatement agents in biomass to bioproducts applications. Biomass Bioenergy.

[17] Ho M.C., Ong V.Z., Wu T.Y. (2019). Potential use of alkaline hydrogen peroxide in lignocellulosic biomass pretreatment and valorization—A review. Renew. Sustain. Energy Rev..

[18] Hou Q., Ju M., Li W., Liu L., Chen Y., Yang Q. (2017). Pretreatment of Lignocellulosic Biomass with Ionic Liquids and Ionic Liquid-Based Solvent Systems. Molecules.

[19] Zhang J., Zhang X., Yang M., Singh S., Cheng G. (2021). Transforming lignocellulosic biomass into biofuels enabled by ionic liquid pretreatment. Bioresour. Technol..

[20] Parawira W., Tekere M. (2011). Biotechnological strategies to overcome inhibitors in lignocellulose hydrolysates for ethanol production: Review. Crit. Rev. Biotechnol..

[21] Tramontina R., Brenelli de Paiva L.B., Sodré V., Franco Cairo J.P., Travália B., Egawa V., Goldbeck R., Squina F. (2020). Enzymatic removal of inhibitory compounds from lignocellulosic hydrolysates for biomass to bioproducts applications. World J. Microbiol. Biotechnol..

[22] Moreno A.D., Ibarra D., Alvira P., Tomas-Pejo E., Ballesteros M. (2015). A review of biological delignification and detoxification methods for lignocellulosic bioethanol production. Crit. Rev. Biotechnol..

[23] Olofsson K., Bertilsson M., Lidén G. (2008). A short review on SSF—An interesting process option for ethanol production from lignocellulosic feedstocks. Biotechnol. Biofuels.

[24] Den Haan R., Van Rensburg E., Rose S., Görgens J., van Zyl W. (2015). Progress and challenges in the engineering of non-cellulolytic microorganisms for consolidated bioprocessing. Curr. Opin. Biotechnol..

[25] Zanellati A., Spina F., Bonaterra M., Dinuccio E., Varese G.C., Scarpeci T.E. (2021). Screening and evaluation of phenols and furans degrading fungi for the biological pretreatment of lignocellulosic biomass. Int. Biodeterior. Biodegrad..

[26] Saldarriaga-Hernandez S., Velasco-Ayala C., Leal-Isla Flores P., de Jesus Rostro-Alanis M., Parra-Saldivar R., Iqbal H.M.N., Carrillo-Nieves D. (2020). Biotransformation of lignocellulosic biomass into industrially relevant products with the aid of fungi-derived lignocellulolytic enzymes. Int. J. Biol. Macromol..

[27] Leite P., Sousa D., Fernandes H., Ferreira M., Costa A.R., Filipe D., Gonçalves M., Peres H., Belo I., Salgado J.M. (2021). Recent advances in production of lignocellulolytic enzymes by solid-state fermentation of agro-industrial wastes. Curr. Opin. Green Sustain. Chem..

[28] Palmqvist E., Hahn-Hägerdal B., Szengyel Z., Zacchi G., Rèczey K. (1997). Simultaneous detoxification and enzyme production of hemicellulose hydrolysates obtained after steam pretreatment. Enzym. Microb. Technol..

[29] Ali S.S., Nugent B., Mullins E., Doohan F.M. (2016). Fungal-mediated consolidated bioprocessing: The potential of *Fusarium oxysporum* for the lignocellulosic ethanol industry. AMB Express.

[30] Nwaefuna A.E., Rumbold K., Boekhout T., Zhou N. (2021). Bioethanolic yeasts from dung beetles: Tapping the potential of extremophilic yeasts for improvement of lignocellulolytic feedstock fermentation. Biotechnol. Biofuels.

[31] López M., Dien B., Moreno J., Bothast R. (2004). Isolation of microorganisms for biological detoxification of lignocellulosic hydrolysates. Appl. Microbiol. Biotechnol..

[32] Ferdeș M., Dincă M.N., Moiceanu G., Zăbavă B.Ș., Paraschiv G. (2020). Microorganisms and Enzymes Used in the Biological Pretreatment of the Substrate to Enhance Biogas Production: A Review. Sustainability.

[33] Shahab R.L., Brethauer S., Davey M.P., Smith A.G., Vignolini S., Luterbacher J.S., Studer M.H. (2020). A heterogeneous microbial consortium producing short-chain fatty acids from lignocellulose. Science.

[34] Singh A., Bedore S.R., Sharma N.K., Lee S.A., Eiteman M.A., Neidle E.L. (2019). Removal of aromatic inhibitors produced from lignocellulosic hydrolysates by Acinetobacter baylyi ADP1 with formation of ethanol by Kluyveromyces marxianus. Biotechnol. Biofuels.

[35] He J., Liu X., Xia J., Xu J., Xiong P., Qiu Z. (2020). One-step utilization of non-detoxified pretreated lignocellulose for enhanced cellulolytic enzyme production using recombinant *Trichoderma reesei* RUT C30 carrying alcohol dehydrogenase and nicotinate phosphoribosyltransferase. Bioresour. Technol..

[36] Aghdam S.A., Brown A.M.V. (2021). Deep learning approaches for natural product discovery from plant endophytic microbiomes. Environ. Microbiome.

[37] Blackwell M. (2011). The fungi: 1, 2, 3... 5.1 million species?. Am. J. Bot..

[38] Suryanarayanan T., Thirunavukkarasu N., Rajulu G., Sasse F., Jansen R., Murali T.S. (2009). Fungal endophytes and bioprospecting. Fungal Biol. Rev..

[39] Gupta S., Chaturvedi P., Kulkarni M.G., Van Staden J. (2020). A critical review on exploiting the pharmaceutical potential of plant endophytic fungi. Biotechnol. Adv..

[40] Suryanarayanan T., Thirunavukkarasu N., Rajulu G., Gopalan V. (2012). Fungal endophytes: An untapped source of biocatalysts. Fungal Divers..

[41] Correa R.C., Rhoden S.A., Mota T.R., Azevedo J.L., Pamphile J.A., de Souza C.G., Polizeli Mde L., Bracht A., Peralta R.M. (2014). Endophytic fungi: Expanding the arsenal of industrial enzyme producers. J. Ind. Microbiol. Biotechnol..

[42] Oses R., Valenzuela S., Freer J., Baeza J., Rodríguez J. (2006). Evaluation of fungal endophytes for lignocellulolytic enzyme production and wood biodegradation. Int. Biodeterior. Biodegrad..

[43] Khan A.L., Al-Harrasi A., Al-Rawahi A., Al-Farsi Z., Al-Mamari A., Waqas M., Asaf S., Elyassi A., Mabood F., Shin J.H. (2016). Endophytic Fungi from Frankincense Tree Improves Host Growth and Produces Extracellular Enzymes and Indole Acetic Acid. PLoS ONE.

[44] Singh E., Sharma S., Dwivedi J., Sharma S. (2012). Diversified potentials of *Ocimum sanctum* Linn (Tulsi):An exhaustive survey. J. Nat. Prod. Plant Resour..

[45] Chowdhary K., Kaushik N. (2015). Fungal Endophyte Diversity and Bioactivity in the Indian Medicinal Plant *Ocimum sanctum* Linn. PLoS ONE.

[46] Ezra D., Hess W., Strobel G. (2005). New endophytic isolates of *Muscodor albus*, a volatile-antibiotic-producing fungus. Microbiology (Reading, England).

[47] Prabha T., Revathi K., Vinod M., Shanthakumar S.P., Bernard P. (2013). A simple method for total genomic DNA extraction from water moulds. Curr. Sci..

[48] Schoch C.L., Seifert K.A., Huhndorf S., Robert V., Spouge J.L., Levesque C.A., Chen W. (2012). Nuclear ribosomal internal transcribed spacer (ITS) region as a universal DNA barcode marker for Fungi. Proc. Natl. Acad. Sci. USA.

[49] Nilsson R.H., Larsson K.-H., Taylor A.F.S., Bengtsson-Palme J., Jeppesen T.S., Schigel D., Kennedy P., Picard K., Glöckner F.O., Tedersoo L. (2018). The UNITE database for molecular identification of fungi: Handling dark taxa and parallel taxonomic classifications. Nucleic Acids Res..

[50] Katoh K., Standley D.M. (2013). MAFFT Multiple Sequence Alignment Software Version 7: Improvements in Performance and Usability. Mol. Biol. Evol..

[51] Kumar S., Stecher G., Li M., Knyaz C., Tamura K. (2018). MEGA X: Molecular Evolutionary Genetics Analysis across Computing Platforms. Mol. Biol. Evol..

[52] Teather R.M., Wood P.J. (1982). Use of Congo red-polysaccharide interactions in enumeration and characterization of cellulolytic bacteria from the bovine rumen. Appl. Environ. Microbiol..

[53] Kasana R.C., Salwan R., Dhar H., Dutt S., Gulati A. (2008). A Rapid and Easy Method for the Detection of Microbial Cellulases on Agar Plates Using Gram’s Iodine. Curr. Microbiol..

[54] Haile M., Kang W.H. (2019). Isolation, Identification, and Characterization of Pectinolytic Yeasts for Starter Culture in Coffee Fermentation. Microorganisms.

[55] Mandels M., Reese E.T. (1960). Induction of cellulase in fungi by cellobiose. J. Bacteriol..

[56] Saqib A., Whitney P. (2006). Esculin gel diffusion assay (EGDA): A simple and sensitive method for screening β-glucosidases. Enzym. Microb. Technol..

[57] Navarro D., Rosso M.N., Haon M., Olive C., Bonnin E., Lesage-Meessen L., Chevret D., Coutinho P.M., Henrissat B., Berrin J.G. (2014). Fast solubilization of recalcitrant cellulosic biomass by the basidiomycete fungus *Laetisaria arvalis* involves successive secretion of oxidative and hydrolytic enzymes. Biotechnol. Biofuels.

[58] Sista Kameshwar A., Qin W. (2017). Qualitative and Quantitative Methods for Isolation and Characterization of Lignin-Modifying Enzymes Secreted by Microorganisms. BioEnergy Res..

[59] Magalhães D.B., de Carvalho M.E.A., Bon E., Neto J.S.A., Kling S.H. (1996). Colorimetric assay for lignin peroxidase activity determination using methylene blue as substrate. Biotechnol. Tech..

[60] Brown J., Li D., Alic M., Gold M. (1994). Heat Shock Induction of Manganese Peroxidase Gene Transcription in *Phanerochaete chrysosporium*. Appl. Environ. Microbiol..

[61] Xiros C., Vafiadi C., Paschos T., Christakopoulos P. (2011). Toxicity tolerance of *Fusarium oxysporum* towards inhibitory compounds formed during pretreatment of lignocellulosic materials. J. Chem. Technol. Biotechnol..

[62] Wei W., Xiong Y., Zhu W., Wang N., Yang G., Peng F. (2016). *Colletotrichum higginsianum* Mitogen-Activated Protein Kinase ChMK1: Role in Growth, Cell Wall Integrity, Colony Melanization, and Pathogenicity. Front. Microbiol..

[63] Ghose T.K. (1987). Measurement of cellulase activities. Pure Appl. Chem..

[64] Rai R., Kaur B., Singh S., Falco M., Tsang A., Chadha B. (2016). Evaluation of secretome of highly efficient lignocellulolytic *Penicillium* sp. Dal 5 isolated from rhizosphere of conifers. Bioresour. Technol..

[65] Bailey M., Biely P., Poutanen K. (1992). Interlaboratory Testing of Methods for Assay of Xylanase Activity. J. Biotechnol..

[66] Miller G.L. (1959). Use of Dinitrosalicylic Acid Reagent for Determination of Reducing Sugar. Anal. Chem..

[67] Gao L., Gao F., Dongyuan Z., Zhang C., Wu G., Shulin C. (2013). Purification and characterization of a new β-glucosidase from *Penicillium piceum* and its application in enzymatic degradation of delignified corn stover. Bioresour. Technol..

[68] Ortiz G.E., Ponce-Mora M.C., Noseda D.G., Cazabat G., Saravalli C., Lopez M.C., Gil G.P., Blasco M., Alberto E.O. (2017). Pectinase production by *Aspergillus giganteus* in solid-state fermentation: Optimization, scale-up, biochemical characterization and its application in olive-oil extraction. J. Ind. Microbiol. Biotechnol..

[69] Arora D.S., Bajwa P. (2001). Comparison of two assay procedures for lignin peroxidase Enzyme Microb. Enzym. Microb. Technol..

[70] Childs R.E., Bardsley W.G. (1975). The steady-state kinetics of peroxidase with 2,2′-azino-di-(3-ethyl-benzthiazoline-6-sulphonic acid) as chromogen. Biochem. J..

[71] Martinez M.J., Ruiz-Duenas F.J., Guillen F., Martinez A.T. (1996). Purification and catalytic properties of two manganese peroxidase isoenzymes from Pleurotus eryngii. Eur. J. Biochem..

[72] Bradford M.M. (1976). A rapid and sensitive method for the quantitation of microgram quantities of protein utilizing the principle of protein-dye binding. Anal. Biochem..

[73] Kiran Kumar A., Parikh B. (2015). Cellulose-degrading enzymes from Aspergillus terreus D34 and enzymatic saccharification of mild-alkali and dilute-acid pretreated lignocellulosic biomass residues. Bioresour. Bioprocess..

[74] Kumar S., Kaushik N. (2013). Endophytic fungi isolated from oil-seed crop Jatropha curcas produces oil and exhibit antifungal activity. PLoS ONE.

[75] Weir B.S., Johnston P.R., Damm U. (2012). The *Colletotrichum gloeosporioides* species complex. Stud. Mycol..

[76] Hyde K.D., Xu J., Rapior S., Jeewon R., Lumyong S., Niego A.G.T., Abeywickrama P.D., Aluthmuhandiram J.V.S., Brahamanage R.S., Brooks S. (2019). The amazing potential of fungi: 50 ways we can exploit fungi industrially. Fungal Divers..

[77] Yin D., Urresti S., Lafond M., Johnston E.M., Derikvand F., Ciano L., Berrin J.-G., Henrissat B., Walton P.H., Davies G.J. (2015). Structure–function characterization reveals new catalytic diversity in the galactose oxidase and glyoxal oxidase family. Nat. Commun..

[78] Jayawardena R., Hyde K., Damm U., Cai L., Liu M., Li X., Zhang W., Zhao W., Yan J.Y. (2016). Notes on currently accepted species of *Colletotrichum*. Mycosphere.

[79] da Silva L.L., Moreno H.L.A., Correia H.L.N., Santana M.F., de Queiroz M.V. (2020). *Colletotrichum*: Species complexes, lifestyle, and peculiarities of some sources of genetic variability. Appl. Microbiol. Biotechnol..

[80] Agrawal R., Verma A., Singhania R.R., Varjani S., Di Dong C., Kumar Patel A. (2021). Current understanding of the inhibition factors and their mechanism of action for the lignocellulosic biomass hydrolysis. Bioresour. Technol..

[81] Du B., Sharma L., Becker C., Chen S.-F., Mowery R., van Walsum G., Chambliss C. (2010). Effect of Varying Feedstock-Pretreatment Chemistry Combinations on the Formation and Accumulation of Potentially Inhibitory Degradation Products in Biomass Hydrolysates. Biotechnol. Bioeng..

[82] van der Pol E., Bakker R., Baets P., Eggink G. (2014). By-products resulting from lignocellulose pretreatment and their inhibitory effect on fermentations for (bio)chemicals and fuels. Appl. Microbiol. Biotechnol..

[83] Huang C., Wu H., Liu Q.P., Li Y.Y., Zong M.H. (2011). Effects of aldehydes on the growth and lipid accumulation of oleaginous yeast *Trichosporon fermentans*. J. Agric. Food Chem..

[84] Chen S.F., Mowery R.A., Castleberry V.A., van Walsum G.P., Chambliss C.K. (2006). High-performance liquid chromatography method for simultaneous determination of aliphatic acid, aromatic acid and neutral degradation products in biomass pretreatment hydrolysates. J. Chromatogr. A.

[85] Klinke H.B., Thomsen A.B., Ahring B.K. (2004). Inhibition of ethanol-producing yeast and bacteria by degradation products produced during pre-treatment of biomass. Appl. Microbiol. Biotechnol..

[86] Ruan Z., Hollinshead W., Isaguirre C., Tang Y., Liao W., Liu Y. (2015). Effects of inhibitory compounds in lignocellulosic hydrolysates on Mortierella isabellina growth and carbon utilization. Bioresour. Technol..

[87] Sarvari Horvath I., Franzen C.J., Taherzadeh M.J., Niklasson C., Liden G. (2003). Effects of furfural on the respiratory metabolism of *Saccharomyces cerevisiae* in glucose-limited chemostats. Appl. Environ. Microbiol..

[88] Slininger P.J., Dien B.S., Kurtzman C.P., Moser B.R., Bakota E.L., Thompson S.R., O’Bryan P.J., Cotta M.A., Balan V., Jin M. (2016). Comparative lipid production by oleaginous yeasts in hydrolyzates of lignocellulosic biomass and process strategy for high titers. Biotechnol. Bioeng..

[89] Feldman D., Kowbel D.J., Glass N.L., Yarden O., Hadar Y. (2015). Detoxification of 5-hydroxymethylfurfural by the *Pleurotus ostreatus* lignolytic enzymes aryl alcohol oxidase and dehydrogenase. Biotechnol. Biofuels.

[90] Rajulu G., Lai L., Murali T.S., Gopalan V., Suryanarayanan T. (2014). Several fungi from fire-prone forests of southern India can utilize furaldehydes. Mycol. Prog..

[91] Wu H.-s., Shen S.-H., Han J.-m., Liu Y.-d., Liu S.-d. (2009). The effect in vitro of exogenously applied p-hydroxybenzoic acid on *Fusarium oxysporum* f. sp niveum. Phytopathol. Mediterr..

[92] Kudahettige Nilsson R., Holmgren M., Madavi B., Nilsson R., Sellstedt A. (2016). Adaptability of Trametes versicolor to the lignocellulosic inhibitors furfural, HMF, phenol and levulinic acid during ethanol fermentation. Biomass Bioenergy.

[93] Alriksson B., Cavka A., Jönsson L. (2011). Improving the fermentability of enzymatic hydrolysates of lignocellulose through chemical in-situ detoxification with reducing agents. Bioresour. Technol..

[94] Zhu J., Yong Q., xu Y., Yu S. (2010). Detoxification of corn stover prehydrolyzate by trialkylamine extraction to improve the ethanol production with *Pichia stipitis* CBS 5776. Bioresour. Technol..

[95] Malamud D.R., Borralho L.M., Panek A.D., Mattoon J.R. (1979). Modulation of cytochrome biosynthesis in yeast by antimetabolite action of levulinic acid. J. Bacteriol..

[96] Guo Z., Olsson L. (2014). Physiological response of *Saccharomyces cerevisiae* to weak acids present in lignocellulosic hydrolysate. FEMS Yeast Res..

[97] Wu H.-S., Wang Y., Zhang C.-Y., Bao W., Ling N., Liu D., Shen Q.-R. (2009). Growth of in vitro *Fusarium oxysporum* f. sp. niveum in chemically defined media amended with gallic acid. Biol. Res..

[98] Wu H., Liu Z.-J., Cai J., Zong M.-H. (2012). Effect of organic acids on the growth and lipid accumulation of oleaginous yeast *Trichosporon fermentans*. Biotechnol. Biofuels.

[99] Roy S., Nuckles E., Archbold D. (2018). Effects of Phenolic Compounds on Growth of *Colletotrichum* spp. In Vitro. Curr. Microbiol..

[100] Adeboye P.T., Bettiga M., Olsson L. (2014). The chemical nature of phenolic compounds determines their toxicity and induces distinct physiological responses in *Saccharomyces cerevisiae* in lignocellulose hydrolysates. AMB Express.

[101] Banerjee G., Car S., Scott-Craig J., Hodge D., Walton J. (2011). Alkaline peroxide pretreatment of corn stover: Effects of biomass, peroxide, and enzyme loading and composition on yields of glucose and xylose. Biotechnol. Biofuels.

[102] Sun Y., Wang Y., Tian C. (2016). bZIP transcription factor CgAP1 is essential for oxidative stress tolerance and full virulence of the poplar anthracnose fungus *Colletotrichum gloeosporioides*. Fungal Genet. Biol. FG B.

[103] Zhu W., Zhou M., Xiong Z., Peng F., Wei W. (2017). The cAMP-PKA Signaling Pathway Regulates Pathogenicity, Hyphal Growth, Appressorial Formation, Conidiation, and Stress Tolerance in *Colletotrichum higginsianum*. Front. Microbiol..

[104] Karatzos S., Edye L., Doherty W. (2012). Sugarcane bagasse pretreatment using three imidazolium-based ionic liquids; Mass balances and enzyme kinetics. Biotechnol. Biofuels.

[105] Yang B., Wyman C. (2008). Pretreatment: The key to unlocking low-cost cellulosic ethanol. Ltd|Biofuels Bioprod. Biorefin..

[106] Xu J., Wang X., Hu L., Xia J., Wu Z., Xu N., Dai B., Wu B. (2015). A novel ionic liquid-tolerant *Fusarium oxysporum* BN secreting ionic liquid-stable cellulase: Consolidated bioprocessing of pretreated lignocellulose containing residual ionic liquid. Bioresour. Technol..

[107] Lima D., Costa T.P.C., Emri T., Pocsi I., Pupin B., Rangel D.E.N. (2021). Fungal tolerance to Congo red, a cell wall integrity stress, as a promising indicator of ecological niche. Fungal Biol..

[108] Oliva J.M., Ballesteros I., Negro M.J., Manzanares P., Cabanas A., Ballesteros M. (2004). Effect of binary combinations of selected toxic compounds on growth and fermentation of *Kluyveromyces marxianus*. Biotechnol. Prog..

[109] Zhang J., Zhu Z., Wang X., Wang N., Wang W., Bao J. (2010). Biodetoxification of toxins generated from lignocellulose pretreatment using a newly isolated fungus, *Amorphotheca resinae* ZN1, and the consequent ethanol fermentation. Biotechnol. Biofuels.

[110] Laluce C., Igbojionu L.I., Silva J.L., Ribeiro C.A. (2019). Statistical prediction of interactions between low concentrations of inhibitors on yeast cells responses added to the SD-medium at low pH values. Biotechnol. Biofuels.

[111] Zhang P.H., Yu X.Y., Weng L.X., Sun L.L., Mao Z.C., Zhang Y.L. (2019). Degradation of Ferulic Acid by the Endophytic Fungus *Colletotrichum gloeosporioides* TMTM-13 Associated with *Ostrya rehderiana* Chun. ACS Omega.

[112] Schnabel G., Tan Q., Schneider V., Ishii H. (2021). Inherent tolerance of *Colletotrichum gloeosporioides* to fludioxonil. Pestic. Biochem. Physiol..

[113] Pompeu G.B., Pietrobon V.C., Andreote C.C.F., Ferreira L.F.R., Aguiar M., Sartori S.B., Cruz S.H., Monteiro R.T.R. (2019). Role of the antioxidant defense system during the production of lignocellulolytic enzymes by fungi. Int. Microbiol..

[114] Dubrulle G., Picot A., Madec S., Corre E., Pawtowski A., Baroncelli R., Zivy M., Balliau T., Le Floch G., Pensec F. (2020). Deciphering the Infectious Process of *Colletotrichum lupini* in Lupin through Transcriptomic and Proteomic Analysis. Microorganisms.

[115] Ribeaucourt D., Saker S., Navarro D., Bissaro B., Drula E., Correia L.O., Haon M., Grisel S., Lapalu N., Henrissat B. (2021). Time-resolved secretome analysis of three *Colletotrichum* species identifies copper radical alcohol oxidases for the production of fatty aldehydes. bioRxiv.

[116] Valiante V. (2017). The Cell Wall Integrity Signaling Pathway and Its Involvement in Secondary Metabolite Production. J. Fungi.

[117] Martinez D., Berka R.M., Henrissat B., Saloheimo M., Arvas M., Baker S.E., Chapman J., Chertkov O., Coutinho P.M., Cullen D. (2008). Genome sequencing and analysis of the biomass-degrading fungus *Trichoderma reesei* (syn. *Hypocrea jecorina*). Nat. Biotechnol..

[118] Liu D., Li J., Zhao S., Zhang R., Wang M., Miao Y., Shen Y., Shen Q. (2013). Secretome diversity and quantitative analysis of cellulolytic *Aspergillus fumigatus* Z5 in the presence of different carbon sources. Biotechnol. Biofuels.

[119] Hernandez-Guzman A., Flores-Martinez A., Ponce-Noyola P., Villagomez-Castro J.C. (2016). Purification and characterization of an extracellular beta-glucosidase from *Sporothrix schenckii*. FEBS Open Bio.

[120] de Almeida M.N., Guimaraes V.M., Bischoff K.M., Falkoski D.L., Pereira O.L., Goncalves D.S., de Rezende S.T. (2011). Cellulases and hemicellulases from endophytic *Acremonium* species and its application on sugarcane bagasse hydrolysis. Appl. Biochem. Biotechnol..

[121] Dey P., Banerjee J., Maiti M.K. (2011). Comparative lipid profiling of two endophytic fungal isolates—*Colletotrichum* sp. and *Alternaria* sp. having potential utilities as biodiesel feedstock. Bioresour. Technol..

[122] Tian F., Xie Z.-l., Zhao L.-z., Guo J., Han X.-b., Xie L.-f., Wang Y., Chang X.-y. (2015). Comparative secretome analysis of *Fusarium* sp. Q7-31T during liquid fermentation using oat straw as a carbon source. Ann. Microbiol..

[123] Acosta-Rodríguez I., Piñón-Escobedo C., Zavala Paramo M., Cano-Camacho H. (2005). Degradation of cellulose by the bean-pathogenic fungus *Colletotrichum lindemuthianum*. Production of extracellular cellulolytic enzymes by cellulose induction. Antonie van Leeuwenhoek.

[124] Amoroso A., Villalobos R., Gonzalez B., Vicuña R. (2009). Hydroquinone and H_2_O_2_ differentially affect the ultrastructure and expression of ligninolytic genes in the basidiomycete *Ceriporiopsis subvermispora*. FEMS Microbiol. Lett..

[125] Castaño J., Zhang J., Anderson C., Schilling J. (2018). Oxidative damage control as a brown rot fungus attacks wood using oxygen radicals. Appl. Environ. Microbiol..

[126] Alves L.F., Meleiro L.P., Silva R.N., Westmann C.A., Guazzaroni M.E. (2018). Novel Ethanol- and 5-Hydroxymethyl Furfural-Stimulated beta-Glucosidase Retrieved From a Brazilian Secondary Atlantic Forest Soil Metagenome. Front. Microbiol..

[127] Larsson S., Quintana-Sáinz A., Reimann A., Nilvebrant N.-O., Jönsson L.J. (2000). Influence of lignocellulose-derived aromatic compounds on oxygen-limited growth and ethanolic fermentation by *Saccharomyces cerevisiae*. Appl. Biochem. Biotechnol..

[128] Elisashvili V., Kachlishvili E., Khardziani T., Agathos S.N. (2010). Effect of aromatic compounds on the production of laccase and manganese peroxidase by white-rot basidiomycetes. J. Ind. Microbiol. Biotechnol..

[129] Suzuki M., Hunt C., Houtman C., Dalebroux Z., Hammel K. (2007). Fungal hydroquinones contribute to brown rot of wood. Environ. Microbiol..

[130] Kracher D., Scheiblbrandner S., Felice A.K., Breslmayr E., Preims M., Ludwicka K., Haltrich D., Eijsink V.G., Ludwig R. (2016). Extracellular electron transfer systems fuel cellulose oxidative degradation. Science.

[131] North M., Tandon V.J., Thomas R., Loguinov A., Gerlovina I., Hubbard A.E., Zhang L., Smith M.T., Vulpe C.D. (2011). Genome-wide functional profiling reveals genes required for tolerance to benzene metabolites in yeast. PLoS ONE.

[132] Tejirian A., Xu F. (2010). Inhibition of cellulase-catalyzed lignocellulosic hydrolysis by iron and oxidative metal ions and complexes. Appl. Environ. Microbiol..

[133] Lucas A., Ao-Ieong E.S.Y., Williams A.T., Jani V.P., Muller C.R., Yalcin O., Cabrales P. (2019). Increased Hemoglobin Oxygen Affinity With 5-Hydroxymethylfurfural Supports Cardiac Function During Severe Hypoxia. Front. Physiol..

[134] Godse R., Bawane H., Tripathi J., Kulkarni R. (2021). Unconventional β-Glucosidases: A Promising Biocatalyst for Industrial Biotechnology. Appl. Biochem. Biotechnol..

[135] Srivastava N., Rathour R., Jha S., Pandey K., Srivastava M., Thakur V.K., Sengar R.S., Gupta V.K., Mazumder P.B., Khan A.F. (2019). Microbial Beta Glucosidase Enzymes: Recent Advances in Biomass Conversation for Biofuels Application. Biomolecules.

