# Supplementary material for: A Lignocellulolytic Colletotrichum sp. OH with Broad-Spectrum Tolerance to Lignocellulosic Pretreatment Compounds and Derivatives and the Efficiency to Produce Hydrogen Peroxide and 5-Hydroxymethylfurfural Tolerant Cellulases
Source: J Fungi (Basel). 2021 Sep 22;7(10):785. doi: 10.3390/jof7100785 (PMC8540663; doi:10.3390/jof7100785)
Supplement: Supplementary file 1 [file jof-07-00785-s001.zip › jof-1338239-supplementary/FigS8.pdf]

Combination	EnG	BGL	XLN	Inhibitor(s)	Biomass Growth (IC50)
C-5-IC75	63.6	134.2	25.9		
C-5-IC50	83.9	129.8	50.8	H_2O_2	45.3
C-5-IC25	124.0	125.2	86.1		
C-2-IC25	91.5	132.3	85.3		
C-2-IC50	60.4	124.3	64.9	HMF	50.7
C-2-IC75	31.9	94.0	31.2		
C-7-IC25	82.5	95.0	75.6		
C-7-IC50	58.8	50.0	53.7	HMF+H_2O_2	43.5
C-7-IC75	31.6	32.0	46.3		
C-6-IC25	76.8	89.5	64.9		
C-6-IC50	48.9	38.5	51.9	HMF+HQ	38.9
C-6-IC75	19.1	30.4	32.6		
C-11-IC25	75.3	52.0	53.0		
C-11-IC50	38.7	31.0	32.8	HMF+HQ+H_2O_2	22.5
C-11-IC75	8.1	8.0	15.0		
C-8-IC25	64.0	63.0	69.9		
C-8-IC50	40.9	38.5	51.0	HMF+LA	45.3
C-8-IC75	21.3	21.2	37.4		
C-9-IC25	61.5	62.5	61.9		
C-9-IC50	41.4	20.0	59.0	HMF+LA+H_2O_2	29.0
C-9-IC75	6.6	3.6	12.9		
C-10-IC25	60.0	45.9	51.3		
C-10-IC50	37.9	34.8	39.1	HMF+LA+HQ	30.8
C-10-IC75	10.0	7.4	7.0		
C-16-IC25	4.4	26.6	39.05		
C-16-IC50	1.4	11.1	19.27	HMF+LA+HQ+H_2O_2	0
C-16-IC75	1.2	1.6	5.53		
C-4-IC25	58.7	59.3	51.3		
C-4-IC50	42.1	34.8	33.2	HQ	57.4
C-4-IC75	0.0	13.1	7.0		
C-15-IC25	62.3	63.5	61.94		
C-15-IC50	38.7	34.8	30.86	HQ+H_2O_2	45.7
C-15-IC75	7.0	5.6	15.56		
C-3-IC25	64.0	91.0	82.6		
C-3-IC50	82.5	38.4	34.2	LA	65.2
C-3-IC75	19.1	13.7	9.6		
C-12-IC25	62.3	61.8	62.4		
C-12-IC50	42.2	40.1	49.9	LA+H_2O_2	46.7
C-12-IC75	14.3	23.0	23.7		
C-13-IC25	59.0	42.3	60.9		
C-13-IC50	39.8	33.5	35.57	LA+HQ	42.0
C-13-IC75	11.3	10.7	19		
C-14-IC25	58.7	52.0	49.9		
C-14-IC50	25.1	26.6	32.76	LA+HQ+H_2O_2	29.0
C-14-IC75	5.0	7.8	9.93		
C-1	100.0	100.0	100.0	NONE	100.0

Figure S8: Relative Enzyme activity of Endoglucanases (EnG), β-glucosidases (BGL), and Xylanases (XLN) in the presence of lignocellulose-derived inhibitor combinations. Three concentrations (IC_{25}, IC_{50}, IC_{75}) of each chemical were tested for enzyme inhibition. The codes in the first column correspond to the respective chemicals in the 5th column. The relative biomass at IC_{50} value is mentioned for comparison. The cells are color-coded with green for highest and red for lowest relative activity. The tolerance is defined at > 50% or more relative activity.
