# Supplementary material for: A Lignocellulolytic Colletotrichum sp. OH with Broad-Spectrum Tolerance to Lignocellulosic Pretreatment Compounds and Derivatives and the Efficiency to Produce Hydrogen Peroxide and 5-Hydroxymethylfurfural Tolerant Cellulases
Source: J Fungi (Basel). 2021 Sep 22;7(10):785. doi: 10.3390/jof7100785 (PMC8540663; doi:10.3390/jof7100785)
Supplement: Supplementary file 1 [file jof-07-00785-s001.zip › jof-1338239-supplementary/FigS9.pdf]

Combinations	Lac	LiP	MnP	Inhibitor(s)
C1	100.0	100.0	100.0	None
C-15-IC25	67.1	62.2	80.4	HQ+H_2O_2
C-5-IC25	61.6	92.2	75.1	H_2O_2
C-15-IC50	36.8	30.2	65.2	HQ+H_2O_2
C-4-IC25	84.5	76.2	65.0	HQ
C-13-IC25	40.4	50.6	62.3	LA+HQ
C-12-IC25	41.6	46.2	60.7	LA+H_2O_2
C-5-IC50	41.1	54.7	57.4	H_2O_2
C-2-IC25	61.6	53.2	54.5	HMF
C-6-IC25	57.1	46.2	50.3	HMF+HQ
C-4-IC50	45.7	42.1	48.4	HQ
C-8-IC25	24.4	25.1	45.1	HMF+LA
C-12-IC50	22.1	18.8	45.1	LA+H_2O_2
C-3-IC25	47.7	53.3	44.3	LA
C-11-IC25	26.3	52.1	44.0	HMF+HQ+H_2O_2
C-13-IC50	20.3	30.6	43.5	LA+HQ
C-10-IC25	22.1	45.6	41.6	HMF+LA+HQ
C-7-IC25	42.9	51.3	32.1	HMF+H_2O_2
C-9-IC25	31.7	31.9	32.0	HMF+LA+H_2O_2
C-11-IC50	12.3	31.7	31.1	HMF+HQ+H_2O_2
C-2-IC50	31.1	24.0	30.4	HMF
C-14-IC25	24.9	42.1	30.4	LA+HQ+H_2O_2
C-3-IC50	31.1	23.4	27.1	LA
C-14-IC50	12.3	26.1	26.2	LA+HQ+H_2O_2
C-8-IC50	10.5	18.3	23.0	HMF+LA
C-6-IC50	24.4	33.2	22.4	HMF+HQ
C-9-IC50	13.5	12.7	22.1	HMF+LA+H_2O_2
C-7-IC50	10.5	35.1	20.1	HMF+H_2O_2
C-10-IC50	10.5	12.3	17.3	HMF+LA+HQ
C-16-IC25	13.0	11.0	9.0	ALL
C-16-IC50	0.0	5.1	0.0	ALL
C-16-IC75	0.0	0.0	0.0	ALL

Figure S9: Relative Enzyme Activity of Laccases (Lac), Lignin peroxidases (LiP), and Manganese peroxidases (MnP) in the presence of lignocellulose-derived inhibitor combinations. Three values of each chemical (IC_{25}, IC_{50}, IC_{75}) were tested for enzyme inhibition. The cells are color-coded with green for highest and red for lowest relative activity. The tolerance is defined at > 50% or more relative activity. For several combinations, the IC_{75} enzyme activities were not detected.
