# Supplementary material for: A Lignocellulolytic Colletotrichum sp. OH with Broad-Spectrum Tolerance to Lignocellulosic Pretreatment Compounds and Derivatives and the Efficiency to Produce Hydrogen Peroxide and 5-Hydroxymethylfurfural Tolerant Cellulases
Source: J Fungi (Basel). 2021 Sep 22;7(10):785. doi: 10.3390/jof7100785 (PMC8540663; doi:10.3390/jof7100785)
Supplement: Supplementary file 1 [file jof-07-00785-s001.zip › jof-1338239-supplementary/Supplementary Figures-list.pdf]

Title: A Lignocellulolytic *Colletotrichum* sp. OH with Broad-Spectrum Tolerance to Lignocellulosic Pretreatment Compounds and Derivatives and the Efficiency to Produce Hydrogen Peroxide and 5-Hydroxymethylfurfural Tolerant Cellulases

Kakoli Chanda[1], Atifa Begum Mozumder[1], Ringhoilal Chorei[1], Ridip Kumar Gogoi[1] & Himanshu Kishore Prasad[1*]

Department of Life Science and Bioinformatics, Assam University, Silchar 788011, India;
kakolimic@gmail.com (K.C.); atifamozumder@gmail.com (A.B.M.);
choreiringhoilal@gmail.com (R.C.);
ridip.gogoi09@gmail.com (R.K.G)
*: Corresponding author: e-mail: himanshu.k.prasad@aus.ac.in.

Figure S1: Uncompressed Rooted Maximum-likelihood phylogenetic tree using s ITS gene sequences

Figure S2: Rooted Neighbor-joining phylogenetic tree based on sequences of LSU gene

Figure S3: Growth of *Colletotrichum* sp. OH on different carbon sources

Figure S4: The growth patterns of *Colletotrichum* sp. OH in different pH (4-13) at 30°C

Figure S5: Cell wall stress-tolerant capability of *Colletotrichum* sp. OH

Figure S6: Growth profile of *Colletotrichum* sp. OH on commercial media and agro-waste at 30^{0}C

Figure S7: Pectinase activity of *Colletotrichum* sp. OH in the presence of 1% citrus pectin

Figure S8: Relative Enzyme activity of Endoglucanases (EnG), β-glucosidases (BGL), and Xylanases (XLN) in the presence of lignocellulose-derived inhibitor combinations

Figure S9: Relative Enzyme Activity of Laccases (Lac), Lignin peroxidases (LiP), and Manganese peroxidases (MnP) in the presence of lignocellulose-derived inhibitor combinations.
