# Supplementary figures and images for: A Lignocellulolytic Colletotrichum sp. OH with Broad-Spectrum Tolerance to Lignocellulosic Pretreatment Compounds and Derivatives and the Efficiency to Produce Hydrogen Peroxide and 5-Hydroxymethylfurfural Tolerant Cellulases
Source: J Fungi (Basel). 2021 Sep 22;7(10):785. doi: 10.3390/jof7100785 (PMC8540663; doi:10.3390/jof7100785)

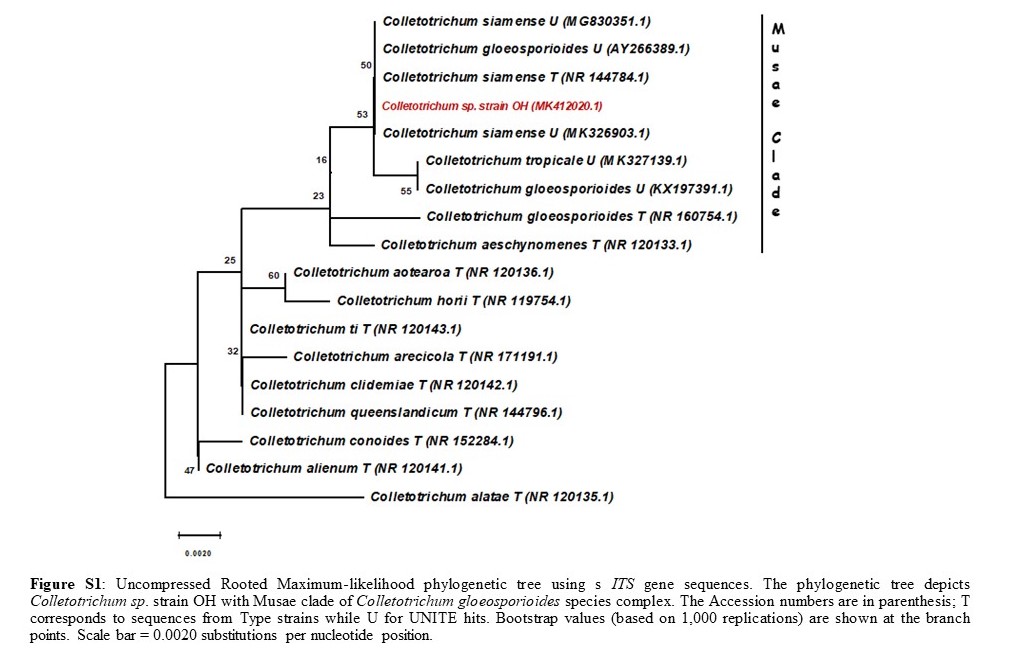

Supplement: Supplementary file 1 [file jof-07-00785-s001.zip › jof-1338239-supplementary/FigS1.jpg]

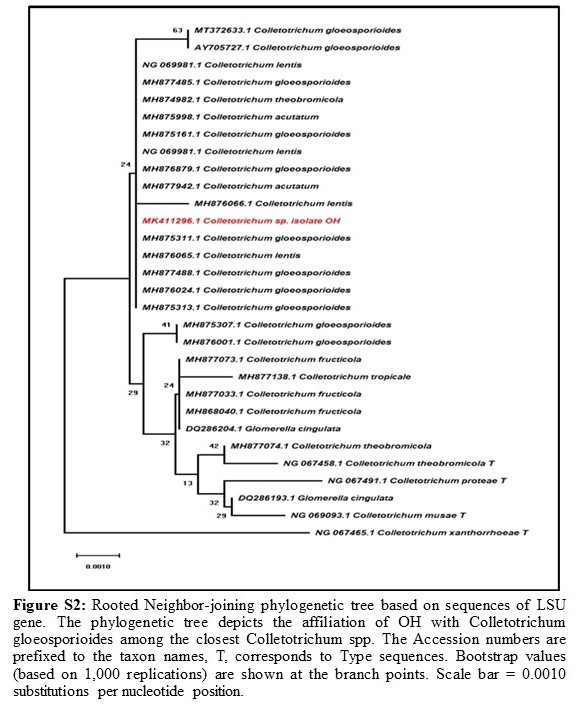

Supplement: Supplementary file 1 [file jof-07-00785-s001.zip › jof-1338239-supplementary/FigS2.jpg]

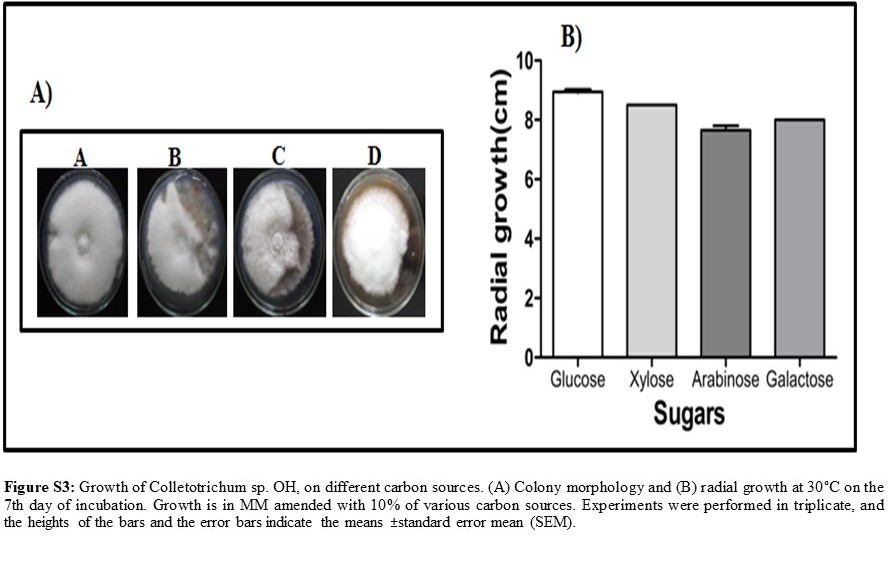

Supplement: Supplementary file 1 [file jof-07-00785-s001.zip › jof-1338239-supplementary/FigS3.jpg]

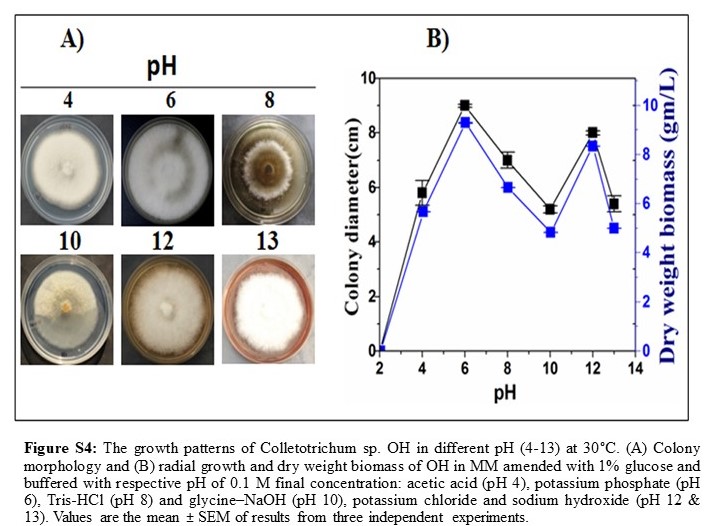

Supplement: Supplementary file 1 [file jof-07-00785-s001.zip › jof-1338239-supplementary/FigS4.jpg]

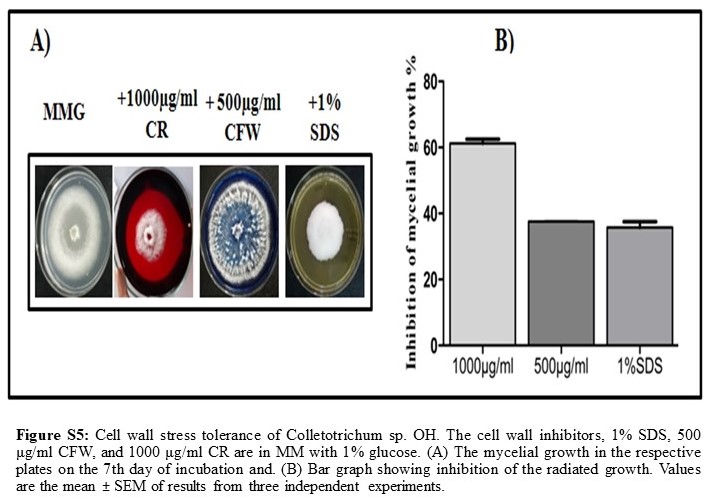

Supplement: Supplementary file 1 [file jof-07-00785-s001.zip › jof-1338239-supplementary/FigS5.jpg]

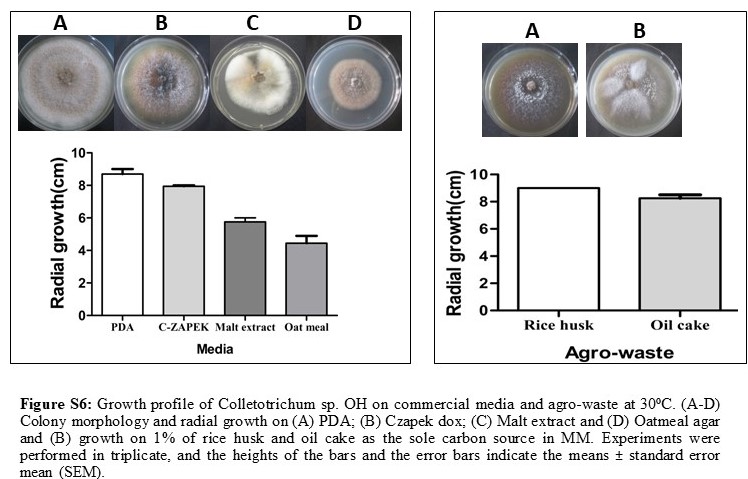

Supplement: Supplementary file 1 [file jof-07-00785-s001.zip › jof-1338239-supplementary/FigS6.jpg]

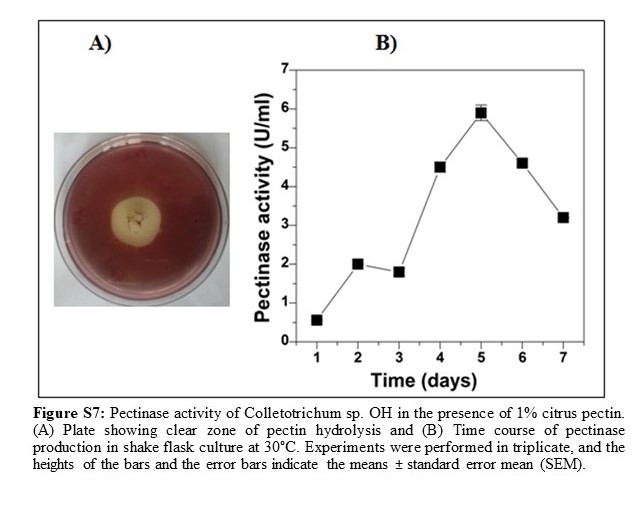

Supplement: Supplementary file 1 [file jof-07-00785-s001.zip › jof-1338239-supplementary/FigS7.jpg]
